# Diacylglycerol kinase and phospholipase D inhibitors alter the cellular lipidome and endosomal sorting towards the Golgi apparatus

**DOI:** 10.1007/s00018-020-03551-6

**Published:** 2020-05-23

**Authors:** Anne Berit Dyve Lingelem, Simona Kavaliauskiene, Ruth Halsne, Tove Irene Klokk, Michal A. Surma, Christian Klose, Tore Skotland, Kirsten Sandvig

**Affiliations:** 1 Department of Molecular Cell Biology, Institute for Cancer Research, The Norwegian Radium Hospital, Oslo University Hospital, Oslo, Norway; 2 Present Address: Department of Forensic Biology, Oslo University Hospital, Oslo, Norway; 3 Present Address: Regional Committees for Medical and Health Research Ethics, University of Oslo, Oslo, Norway; 4 Lipotype GmbH, Dresden, Germany; 5 Department of Biosciences, University of Oslo, Oslo, Norway

**Keywords:** Intracellular transport, CAY10594, R59022, PLD3, SNX2, Vps35

## Abstract

The membrane lipids diacylglycerol (DAG) and phosphatidic acid (PA) are important second messengers that can regulate membrane transport by recruiting proteins to the membrane and by altering biophysical membrane properties. DAG and PA are involved in the transport from the Golgi apparatus to endosomes, and we have here investigated whether changes in these lipids might be important for regulation of transport to the Golgi using the protein toxin ricin. Modulation of DAG and PA levels using DAG kinase (DGK) and phospholipase D (PLD) inhibitors gave a strong increase in retrograde ricin transport, but had little impact on ricin recycling or degradation. Inhibitor treatment strongly affected the endosome morphology, increasing endosomal tubulation and size. Furthermore, ricin was present in these tubular structures together with proteins known to regulate retrograde transport. Using siRNA to knock down different isoforms of PLD and DGK, we found that several isoforms of PLD and DGK are involved in regulating ricin transport to the Golgi. Finally, by performing lipidomic analysis we found that the DGK inhibitor gave a weak, but expected, increase in DAG levels, while the PLD inhibitor gave a strong and unexpected increase in DAG levels, showing that it is important to perform lipidomic analysis when using inhibitors of lipid metabolism.

**Electronic supplementary material:**

The online version of this article (10.1007/s00018-020-03551-6) contains supplementary material, which is available to authorized users.

## Introduction

The cell itself and all of its organelles are surrounded by a lipid bilayer, made up of phospholipids, sphingolipids and sterols. It is becoming increasingly clear that lipids play an active role in numerous cellular processes, including transport events and signaling. Both diacylglycerol (DAG) and phosphatidic acid (PA) are important membrane lipids and second messengers that contribute to cellular processes either by their biophysical effect on the membrane or by recruiting proteins to the membrane. Due to their small head groups, DAG and PA have conical shapes, and local accumulation of DAG or PA can impart negative curvature to membranes thereby facilitating membrane budding, and fusion- and fission reactions during vesicular transport [1]. DAG- or PA-mediated protein recruitment and activation of kinases can also contribute to modulation of vesicular trafficking [2]. DAG interacts with proteins containing a C1-domain, such as the protein kinase C (PKC) and protein kinase D (PKD) families [3]. A broad range of PA-binding proteins have been identified [4, 5]. PA-protein binding is thought to be mediated by interaction between positively charged amino acids in target proteins and the negatively charged head group of PA, but other factors, such as the membrane packing and curvature, are also important determinants for PA-protein interaction [6].

PA and DAG can be formed by de novo lipid biosynthesis, and are intermediates for the production of all the glycerophospholipids [1, 7]. DAG and PA are also produced during receptor signaling which activates a number of different signaling pathways, and the levels of DAG and PA must therefore be strictly controlled. DAG and PA are produced by phospholipid hydrolysis mediated by phospholipase C (PLC) and phospholipase D (PLD), respectively. Sequence analyses have revealed six mammalian PLD isoforms, but only three (PLD1, PLD2 and PLD6, also called mitoPLD) have so far been shown to have enzymatic activity resulting in PA production [8]. Importantly it was recently demonstrated that PLD3 is localized on endosomal structures that are positive for retromer components; it plays a role for intracellular sorting and it causes changes in accordance with the idea that it has enzymatic activity [9]. DAG and PA signaling are terminated by the action of DAG kinases (DGKs) and PA phosphatases, respectively, interconverting the two lipids. In mammalian cells, 10 DGK isoforms have been identified, with different tissue expression and subcellular localization [10]. DAG is also formed as a byproduct in sphingomyelin synthesis while PA can also be formed from lysophosphatidic acid (LPA) by the action of LPA acyltransferase (LPAAT).

Protein toxins can be used as tools to study intracellular transport and both the bacterial toxin Shiga toxin (an AB5 toxin), as well as the plant toxin ricin (an AB toxin), have during the years proven useful to discover pathways in cells (for reviews, see [11–13]). The ricin B-chain binds to N-terminal galactoses on glycoproteins and glycolipids on the cell membrane and is therefore likely to be transported along the various pathways that exist in cells. Actually, in 1979, ricin was used to demonstrate that endocytosed molecules could be recycled [14], and some of the early evidence for clathrin-independent endocytosis was obtained using ricin [15, 16]. The ricin A-chain has enzymatic activity that removes a specific adenine from the 60 s ribosomal subunit, thereby preventing protein synthesis. To reach its cellular target, ricin must be transported from endosomes, via the Golgi apparatus, to the endoplasmic reticulum (ER). Here, the A chain is released from the B-chain and translocated into the cytosol where it exerts its toxic action.

In the present study we have examined how modulation of the DAG and PA balance affects retrograde transport. We have found that inhibitors of DGK and PLD strongly increase ricin transport from the endosomes to the Golgi and as a consequence of this upregulation, more toxin is able to reach the cytosol. To study the different steps we have used native ricin as well as ^125^I-labeled ricin and genetically modified ricin that can be sulfated in the Golgi apparatus and mannosylated in the ER [17]. Lipidomic analysis showed that DGK inhibition as expected gave an increase in DAG levels, whereas PLD inhibition surprisingly increased the levels of both DAG and phosphatidylglycerol (PG), a lipid which has so far not been extensively studied [18] and where specific roles in intracellular transport has not, to our knowledge, been reported.

## Materials and methods

### Reagents, antibodies and plasmids

Chemicals were purchased from Sigma-Aldrich unless stated otherwise. Ricin holotoxin was purchased from Sigma-Aldrich. Ricin-sulf1 and ricin-sulf2 were prepared as previously described [19]. The plasmids expressing StxB-sulf2 and the Shiga toxin 1 mutant (Stx1m) were kind gifts from Prof. B Goud (Institut Curie, Paris, France) and Prof. A. D. O’Brien (Uniformed Services University of the Health Sciences, Bethesda, MD, USA), respectively, and StxB-sulf2 [20] and Stx1m [21] were purified as previously described. H_2_^35^SO_4_ (S-RA-1) was from Hartmann Analytic. The iodine-125 radionuclide (^125^I, NEZ033A010MC) for radioactive protein labeling, l-[3,4,5-^3^H(N)]-leucine (NET460005MC) and d-[2-^3^H(N)]-mannose (NET570A005MC) were from Perkin Elmer. The following compounds were used: R59022 (D5919), R59949 (D5794), Bisindolylmaleimide I hydrochloride (BIM I; B6292), Phorbol 12-myristate 13-acetate (PMA; 79346) and Swainsonine (S9263) from Sigma-Aldrich, CAY10593 (13206-10) and CAY10594 (13207-10) from Cayman Chemical, FIPI (3600) and VU 0364739 hydrochloride (4171) were from Tocris, Sotrastaurin (S2791) and SAR405 (S7682) from Selleckchem. The environment-sensitive probe NR12S was a kind gift from Prof. A. Klymchenko (University of Strasbourg, Strasbourg, France). The following Ambion Silencer® Select siRNAs were purchased from Thermo Fisher Scientific: Negative Control No. 1 (4390844), DGKA (4427038 s3913), DGKD (4427037 s224987), DGKE (4427038 s16208), DGKH (4427037 s46229), DGKZ (4427037 s16205), PLD1 (4427038 s10638), PLD2 (4427038 s10642), PLD3 (4427037 s24272).

The following antibodies were used: homemade rabbit anti-ricin antibody, mouse anti-Golgin-97 (A-21270, Molecular Probes), mouse anti-Shiga toxin 1 (STX1-3C10, Toxin Technology), rabbit anti-Golgin-97 (13192S, Cell Signaling Technology), rabbit anti-EEA1 (2411, Cell Signaling Technology), rabbit anti-Phospho-(Ser/Thr) PKD substrate (4381, Cell Signaling Technology), rabbit anti-cofilin (ab42824, Abcam), goat anti-Vps35 (ab10099, Abcam), mouse anti-SNX2 (611308, BD Transduction Laboratories). Secondary antibodies for immunofluorescence were from Jackson ImmunoResearch and Molecular Probes, IRDye secondary antibodies for Western blotting were from LICOR Biosciences, GmbH. Ricin holotoxin was labeled with Alexa Fluor 555 using the Alexa Fluor™ 555 Microscale Protein Labeling Kit (A30007, Invitrogen) according to the manufacturer’s protocol.

### Cell culture

HEp-2 cells (human cervical adenocarcinoma, CCL-23, ATCC), and U-2 OS cells (human bone osteosarcoma, HTB-96, ATCC) were grown in Dulbecco’s Modified Eagle Medium (DMEM, D0819, Sigma-Aldrich) with 10% fetal bovine serum (FBS, F7524, Sigma-Aldrich). PC-3 cells (human prostate adenocarcinoma, CRL-1435, ATCC) were grown in DMEM/F-12 (1:1) (31331–093, Thermo Fisher Scientific) with 7% FBS. CaCo2 cells (human colorectal adenocarcinoma, HTB-37, ATCC) were grown in DMEM with 15% FBS. hTERT-RPE1 cells (retinal pigment epithelia, CRL-4000, ATCC) stably expressing GFP-WDFY2 [22] was a kind gift from Prof. H. Stenmark, Oslo, and were grown in DMEM/F-12 (1:1) with 10% FBS. All cell lines were maintained in medium supplemented with 100 U/ml penicillin and 100 µg/ml streptomycin (P4333, Sigma-Aldrich) at 37 °C and 5% CO_2_.

### siRNA protein knockdown

HEp-2 cells were plated at 1 × 10^5^ cells/well (6 well plate) or 2 × 10^4^ cells/well (24 well plate) the day before transfection. Cells were transfected with Ambion Silencer® Select siRNAs of each target gene or control at a concentration of 10 nM using Lipofectamine® RNAiMAX Transfection Reagent (13778, Thermo Fisher Scientific) according to the manufacturer’s protocol. The transfection medium was replaced with growth medium after 24 h, and assays were carried out 48 h post transfection. Knockdown efficiencies were determined by measuring mRNA levels using qPCR.

### RNA isolation, cDNA synthesis and qPCR

Total RNA was isolated using the RNeasy Plus Mini Kit (74134, Qiagen) according to the manufacturer’s protocol. cDNA was synthesized from 1 µg RNA using the iScript cDNA synthesis kit (1708891, Bio-Rad Laboratories Inc) according to the manufacturer’s protocol. qPCR was performed using the LightCycler® 480 SYBR Green I Master kit (04707516001, Roche Diagnostics) in combination with QuantiTect Primer Assays (all from Qiagen) for DGKA (QT00091112), DGKD (QT00068894), DGKE (QT00090720), DGKH (QT00092939), DGKQ (QT00005348), DGKZ (QT00071176), PLD1 (QT00085512), PLD2 (QT00017682), PLD3 (QT00029239) and TBP (reference gene; QT00000721). Serial dilutions of control samples were used to plot standard curves and to quantify primer efficiencies in each qPCR run. The qPCR was carried out using a LightCycler® 480 Instrument (Roche Diagnostics). The reactions were run in duplicates, and the samples were first denatured at 95 °C for 10 min, followed by 45 cycles of denaturation at 95 °C for 10 s, primer annealing at 55 °C for 20 s and primer extension at 72 °C for 20 s. Upon completion of the cycling steps, a melting curve analysis was done. Cp values and primer efficiencies were determined using the LightCycler® 480 software (Roche Diagnostics).

### Sulfation experiments

For sulfation experiments, the following cell numbers were seeded in 6 well plates one or two days prior to experiments: HEp-2: 2–2.5 × 10^5^ cells/well (1 day) and 1 × 10^5^ cells/well (knockdown experiments), U-2 OS: 2 × 10^5^ cells/well (1 day), PC-3: 3 × 10^5^ cells/well (1 day), CaCo-2: 2 × 10^5^ cells/well (2 days), RPE GFP-WDFY2: 2 × 10^5^ cells/well (1 day). Cells were washed twice with sulfate-free medium (1.0 mM CaCl_2_, 5.4 mM KCl, 1.0 mM MgCl_2_, 0.12 M NaCl, 26.2 mM NaHCO_3_, 10.9 mM NaH_2_PO_4_, 1 g/l D-glucose, 0.01 g/l phenol red, supplemented with MEM vitamin solution, MEM amino acids and MEM non-essential amino acid; pH 7.4) and subsequently incubated with 0.1–0.2 mCi/ml ^35^SO_4_^2−^ for 2 h at 37 °C, before inhibitors were added and the incubation continued for 1 h. For the knockdown experiments, the cells were incubated with 0.2 mCi/ml ^35^SO_4_^2−^ for 3 h. Subsequently, 4 µg/ml ricin-sulf1, 15 µg/ml ricin-sulf2 or 2 µg/ml StxB-sulf2 was added for 1.5 h, 3 h or 1 h, respectively. Samples treated with ricin-sulf1 or ricin-sulf2 were washed twice with 0.1 M lactose in HEPES-buffered medium (MEM without sodium bicarbonate supplemented with 20 mM HEPES, 2 mM l-alanyl-l-glutamine, 100 U\ml penicillin and 100 µg/ml streptomycin) for 5 min at 37 °C to remove surface-bound ricin, then washed with ice-cold PBS (1.1 mM NaH_2_PO_4_, 5.5 mM Na_2_HPO_4_, 138.6 mM NaCl; pH 7.4) and lysed in lysis buffer (0.1 M NaCl, 10 mM Na_2_HPO_4_, 1 mM EDTA, 1% Triton X-100, supplemented with cOmplete™ Protease inhibitor Coctail (05056489001, Roche Diagnostics) and 60 mM *n*-octyl-β-glucopyranoside; pH 7.4). Samples treated with StxB-sulf2 were washed with PBS and lysed. Lysates were cleared by centrifugation and ricin-sulf1, ricin-sulf2 or StxB-sulf2 were immunoprecipitated overnight at 4 °C from cleared lysates using Protein A Sepharose® beads (17-0963-03, GE Healthcare) with the appropriate antibody adsorbed. The immunoprecipitate was washed twice with 0.35% TritonX-100 in PBS, resuspended in Laemmli sample buffer (161–0747, Bio-Rad Laboratories Inc) with 100 mM DTT and boiled for 5 min. The immunoprecipitate was separated by SDS-PAGE, blotted onto a PVDF membrane and visualized by digital autoradiography using a phosphor imaging screen (Imaging Screen-K (Kodak), Bio-Rad Laboratories Inc) and the Molecular Imaging PharosFX system (Bio-Rad Laboratories Inc). Band intensities were quantified using the Quantity One 1-D Analysis Software (Bio-Rad Laboratories Inc). To determine the total amount of protein sulfation, proteins from the supernatants after immunoprecipitation were precipitated with 5% trichloroacetic acid (TCA; 100807, Merck). The TCA precipitate was dissolved in 0.1 M KOH and measured by liquid scintillation counting using a Tri-Carb 2100TR Liquid Scintillation Analyzer (Packard).

### Ricin endocytosis

Ricin was ^125^I-labelled using Pierce Iodination Tubes (cat. no 28601, Thermo Fisher Scientific) according to the manufacturer’s protocol. HEp-2 cells were seeded at a concentration of 5 × 10^4^ cells/well (for inhibitor treatment) or 2 × 10^4^ cells/well (for knockdown) in a 24 well plate one day prior to the experiment. For the treatment with inhibitors, the cells were washed once with HEPES-buffered medium before being incubated with inhibitors for 1 h at 37 °C. Subsequently, ~ 60 ng/ml of ^125^I-labeled ricin was added and the incubation continued for 20 min at 37 °C. The cells were incubated with or without 0.1 M lactose in HEPES-buffered medium for 5 min to remove surface-bound ricin and then washed three times with PBS or 0.1 M lactose. The cells were dissolved in 0.1 M KOH and the radioactivity in the solution was determined by gamma-counting using a Hidex Automatic Gamma Counter (Hidex). Ricin endocytosis was expressed as the amount of ^125^I-labeled ricin in lactose-washed cells divided by the amount of ^125^I-labeled ricin in PBS-washed cells.

### Ricin degradation and recycling

HEp-2 cells were seeded at a concentration of 5 × 10^4^ cells/well in a 24 well plate one day prior to the experiment. The cells were washed once with HEPES-buffered medium before being incubated with inhibitors for 1 h at 37 °C. Then, ~ 60 ng/ml of ^125^I-labeled ricin was added and the incubation was continued for 20 min at 37 °C. Surface-bound ^125^I-labeled ricin was removed by incubating the cells with 0.1 M lactose in HEPES-buffered medium for 5 min and washing three times with the same solution. The ^125^I-labeled ricin was then chased in the presence of inhibitors for 2 h at 37 °C in 1 mM lactose in HEPES-buffered medium to prevent further uptake of the toxin. To determine the amount of degraded and recycled ^125^I-labeled ricin, the medium was collected and proteins were precipitated with a final concentration of 4% TCA and 0.4 mg/ml bovine serum albumin (BSA) and pelleted. The supernatant contains ^125^I released from cells after ^125^I-labeled ricin degradation, whereas the pellet contains recycled ^125^I-labeled ricin. To determine the amount of cell-associated ^125^I-ricin, cells were dissolved in 0.1 M KOH. The amount of ^125^I in the supernatant, pellet and cells was measured by gamma-counting and divided by the total count to give the amount of degradation, recycling and cell association, respectively.

### Immunofluorescence and live cell imaging

#### Microscopes

Images were acquired using Zeiss LSM 780 or LSM 880 laser scanning confocal microscopes equipped with an Ar-Laser Multiline (458/488/514 nm), a DPSS-561 10 (561 nm), a Laser diode 405–30 CW (405 nm), and a HeNe-laser (633 nm); the objective used was Zeiss plan-Apochromat 63x/1.40 Oil DIC M27 (all from Carl Zeiss MicroImaging GmbH).

#### Endosome size analysis

RPE-GFP-WDFY2 cells were seeded onto coverslips immersed in growth media-filled wells. Next day, the cells were washed once with warm HEPES buffered medium and incubated with indicated inhibitors in HEPES buffered medium for 1 h at 37 °C. The cells were fixed in 4% formaldehyde/PBS solution for 20 min at room temperature. Finally, the coverslips were washed 3 times in PBS, rinsed with water and mounted to microscope slides using ProLong™ Diamond antifade mountant (ThermoFisher Scientific). Images were acquired using Zeiss LSM 780 or LSM 880 microscopes with pixel size 0.071 µm and a pinhole of 50 µm. The focus was set to the plane with the majority of the large WDFY2-positive endosomes. Image pre-processing was performed using Fiji software [23]. The background was subtracted using rolling ball with the size 50 and then images were exported to HDF5 format using the Ilastik plugin. Endosome size was analyzed using Ilastik software [24] using the pipeline for the pixel classification followed by object classification (Fig. 6d). The training was performed using 1–2 images from each condition. First, the software was trained to classify pixels based on GFP signal: negative (background), GFP-positive or lumen (within the lumen of large endosomes). Then, GFP-positive pixels were further classified into different objects: small, medium, large/cluster and giant/cluster. This classification failed to discriminate between large endosomes and the clusters, thus it was used only for quantifying small endosomes (GFP-positive structures without lumen). To quantify medium and large endosomes (with visible lumen), pixels classified as luminal in the pixel classification step were used for the object classification. The luminal pixels were separated into three object classes: medium, large and giant. Finally, to quantify cell-covered area in each image, a separate pixel classification was performed on the same images and the pixels were classified as positive (cells; included both strong WDFY2-GFP staining on the endosomes and the weak WDFY2-GFP staining in the cytosol) or negative for WDFY2-GFP signal.

#### Tubule length analysis

RPE-GFP-WDFY2 cells were grown and treated with the inhibitors as described in the section “Endosome size analysis”. To preserve tubules during cell fixation, 16% formaldehyde (18814, Polysciences) was warmed to 37 °C and added drop-wise directly to the cell medium to a final concentration of 4%. The cells were then fixed for 20 min at room temperature, washed three times with PBS and the coverslips were mounted to microscope slides using ProLong™ Diamond antifade mountant. The images were acquired using similar settings as for the “Endosome size analysis”, but using 3D scanning with the step size of 0.37 µm. Images of the maximum intensity projections from the 3D scans were used to analyze the number and length of the tubules in the cells. The number of the tubules per cell and the length of the tubules were quantified by manually marking and measuring all the visible tubules using Fiji software [23]. For the cells that had no visible tubular structures, a value of “0” was added to the measured tubule length. Because it was difficult to distinguish between short tubules and small elongated endosomes, only tubules of at least 0.7 µm lengths (called long tubular structures) were included in the counting for mean length and number of long tubules per cell.

#### Live cell imaging

RPE-GFP-WDFY2 cells were seeded into glass-bottom microwell dishes (MatTek Corporation). Next day, the cells were washed twice with FluoroBrite™ DMEM medium (Gibco™, Thermo Fisher Scientific) and incubated with indicated inhibitors prepared in FluoroBrite™ DMEM medium. The imaging was started after 1 h and continued for up to 30 min. The images were acquired with a Zeiss LSM 880 microscope using Airyscan detector in a fast scanning mode. The images were taken each 2 s for 2 min and processed using ZEN 2.3 Blue software (Carl Zeiss).

#### Immuno staining

For toxin transport to the Golgi and endosome size based on EAA1 staining, U-2 OS or HEp-2 cells grown on coverslips were treated as indicated in figure legends and subsequently fixed in 4% formaldehyde/PBS for 20 min at room temperature, followed by permeabilization in 0.1% Triton X-100 for 5 min and blocking in 5% FBS in PBS for 30 min. The samples were labeled with primary antibodies in 5% FBS for 1 h and with secondary antibodies in 5% FBS for 30 min, with 3 × 5 min wash in PBS after each antibody labeling. Then the coverslips were rinsed with water and mounted to microscope slides using ProLong™ Gold or ProLong™ Diamond antifade mountant with DAPI (ThermoFisher Scientific). Images were acquired using a Zeiss LSM 780 microscope with pixel size 0.071 µm and the pinhole of 50 µm. Individual cells were manually marked in the images and the toxin labeling intensity within the Golgin-97-positive structures was analyzed using Fiji software [23]. To determine the size of EEA1-positive structures, individual cells were marked and the endosome area was measured using the particle analyzer in the Fiji software [23].

For analyzing ricin transport via the endosomal system, RPE-GFP-WDFY2 cells were grown and treated with the inhibitors as described in “Endosome size analysis”. Ricin-Alexa555 was then added to the cell medium (final conc. 1 µg/ml). After 20 min, lactose solution was added to the cell medium (final conc. 0.1 M) to remove surface exposed ricin-Alexa555. After 10 min, the cells were fixed by adding 16% paraformaldehyde (37 °C) directly to the cell medium (final conc. 4%) and incubated for 20 min at room temperature. The cells were washed 3-times with PBS and then permeabilized and blocked using 0.05% saponin/5% FBS in PBS (blocking solution) for 20 min at room temperature. The antibodies were prepared in blocking solution, and the samples were labeled with primary antibodies for 1 h and with the secondary antibodies for 30 min, with 3 × 5 min wash in 0.05% saponin in PBS after each antibody labeling. Then the coverslips were rinsed with water and mounted to microscope slides using ProLong™ Diamond antifade mountant. Images were acquired with a Zeiss LSM 880 microscope using Airyscan detector with a voxel size of 0.035 × 0.035 × 0.16 µm for high resolution imaging and super-resolution processing. Image acquisition, processing and analysis were performed with ZEN 2.3 Blue software (Carl Zeiss MicroImaging GmbH), while 3D visualization of z-stacks was done using Imaris 9.2.1 (Bitplane AG).

### Western blot

HEp-2 cells were seeded at a concentration of 2.5 × 10^5^ cells/well in 6 well plates one day prior to the experiment. The cells were washed and serum-starved in sulfate-free medium for 2 h at 37 °C to mimic the conditions of the sulfation experiments. Inhibitors were added and the incubation continued for 1 h at 37 °C. The cells were washed in TBS (20 mM Tris, 150 mM NaCl, pH 7.6) and lysed in lysis buffer supplemented with PhosStop (04906837001, Roche Diagnostics). Cleared lysates were mixed with Laemmli sample buffer supplemented with 100 mM M DTT and boiled for 5 min, before proteins were separated by SDS-PAGE. Proteins were transferred onto a low-fluorescent PVDF membrane that was subsequently blocked in 5% skim milk in TBS for 40–60 min. The membrane was washed in 0.1% TBS-Tween (TBST), cut and incubated with primary antibodies in 5% BSA in TBST overnight at 4 °C. The membrane was washed and incubated with secondary antibodies in 5% BSA in TBST for 1 h in the dark. After washing and drying, proteins were visualized by scanning the membrane on an Odyssey imaging system (LICOR Biosciences, GmbH). Band intensities were quantified using the Quantity One 1-D Analysis Software (Bio-Rad Laboratories Inc.).

### Mass spectrometry lipidomics

#### Sample preparation

HEp-2 cells were seeded at a concentration of 1.2 × 10^6^ in 10 cm dishes one day prior to experiments. The cells were washed with HEPES-buffered medium and incubated with inhibitors for 1 or 3 h at 37 °C. The cells were then washed with Dulbecco’s PBS without Ca^2+^ and Mg^2+^ (14190094, Thermo Fisher Scientific) and detached with Accutase (A6964, Sigma-Aldrich) at 4 °C. The cells were washed twice in PBS and counted using a Countess II Automated Cell Counter (Thermo Fisher Scientific). The cell concentration was adjusted to a final concentration of 1.5 × 10^6^ cells/ml and samples were stored at -80 °C until shipment to Lipotype GmbH for analysis using mass spectrometry (MS).

#### Lipid extraction for MS lipidomics

MS-based lipid analysis was performed by Lipotype GmbH (Dresden, Germany) as described [25]. Lipids were extracted using a two-step chloroform/methanol procedure [26]. Samples were spiked with internal lipid standard mixture containing: cardiolipin 16:1/15:0/15:0/15:0 (CL), ceramide 18:1;2/17:0 (Cer), diacylglycerol 17:0/17:0 (DAG), hexosylceramide 18:1;2, 12:0 (HexCer), lyso-phosphatidate 17:0 (LPA), lyso-phosphatidylcholine 12:0 (LPC), lyso-phosphatidylethanolamine 17:1 (LPE), lyso-phosphatidylglycerol 17:1 (LPG), lyso-phosphatidylinositol 17:1 (LPI), lyso-phosphatidylserine 17:1 (LPS), phosphatidate 17:0/17:0 (PA), phosphatidylcholine 17:0/17:0 (PC), phosphatidylethanolamine 17:0/17:0 (PE), phosphatidylglycerol 17:0/17:0 (PG), phosphatidylinositol 16:0/16:0 (PI), phosphatidylserine 17:0/17:0 (PS), cholesterol ester 20:0 (CE), sphingomyelin 18:1;2/12:0;0 (SM), triacylglycerol 17:0/17:0/17:0 (TAG). After extraction, the organic phase was transferred to an infusion plate and dried in a speed vacuum concentrator. 1st step dry extract was re-suspended in 7.5 mM ammonium acetate in chloroform/methanol/propanol (1:2:4, v:v:v) and 2nd step dry extract in 33% ethanol solution of methylamine in chloroform/methanol (0.003:5:1; v:v:v). All liquid handling steps were performed using Hamilton Robotics STARlet robotic platform with the Anti Droplet Control feature for organic solvents pipetting.

#### MS data acquisition

Samples were analyzed by direct infusion of a QExactive mass spectrometer (Thermo Fisher Scientific) equipped with a TriVersa NanoMate ion source (Advion Biosciences). Samples were analyzed in both positive and negative ion modes with a resolution of *R*_m/z=200_ = 28,000 for MS and *R*_m/z=200_ = 17,500 for MSMS experiments, in a single acquisition. MSMS was triggered by an inclusion list encompassing corresponding MS mass ranges scanned in 1 Da increments [27]. Both MS and MSMS data were combined to monitor CE, DAG and TAG ions as ammonium adducts; PC, PC-O (ether-linked PC; alkyl or alkenyl), as acetate adducts; and CL, PA, PE, PE-O (ether-linked PE; alkyl or alkenyl), PG, PI and PS as deprotonated anions. MS only was used to monitor LPA, LPE, LPE-O, LPI and LPS as deprotonated anions; Cer, HexCer, SM, LPC and LPC-O as acetate adducts.

#### Data analysis and post-processing

Data were analyzed with in-house developed lipid identification software based on LipidXplorer [28, 29]. Data post-processing and normalization were performed using an in-house developed data management system. Only lipid identifications with a signal-to-noise ratio > 5, and a signal intensity fivefold higher than in corresponding blank samples were considered for further data analysis. The acquired data was then post-processed to remove minor species and possible miss-detections based on these criteria: (1) keep only species that are above 0 in at least 9 samples and are above 0 in both biological replicates in at least 2 conditions; (2) remove all species in the whole sample if there is a clear misdetection (completely different species distribution compared to the rest of the samples). The remaining lipid values were used for figure plotting and further data quantification. Glycerolipid species are listed with the fatty acyl groups separated with a hyphen as the *sn*-position of the fatty acids was not identified.

### Statistical data analysis

The differences between means for two groups were determined using two-tailed Student’s *t*-test. The level of significance was set as follows: *p ≤ 0.05, ** p ≤ 0.01, *** p ≤ 0.005 compared to control unless otherwise stated.

## Results

### Inhibition of DGK and PLD increases retrograde transport to the Golgi

**Fig. 1:**
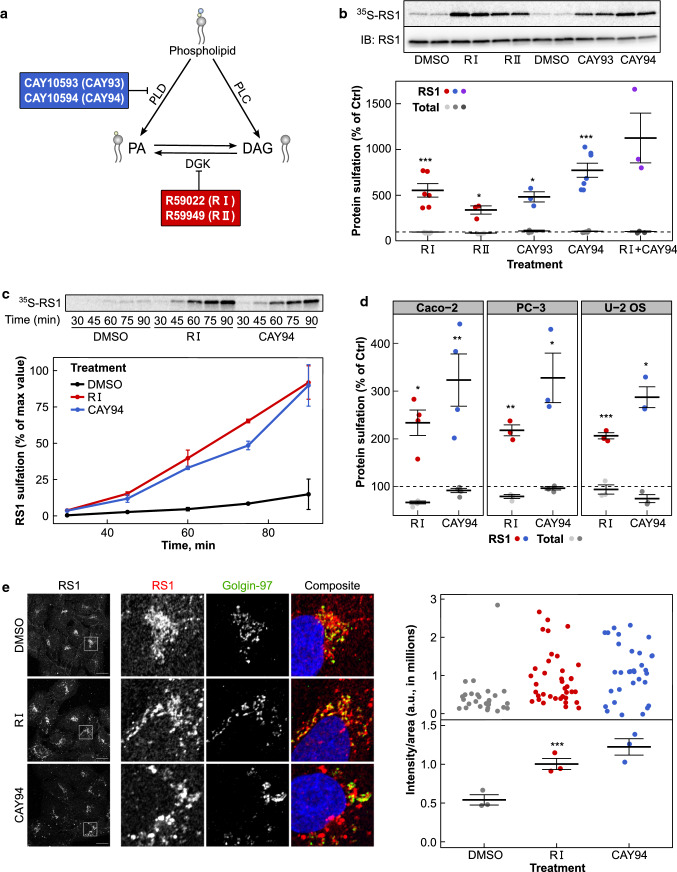
Ricin transport to the Golgi is strongly increased upon DGK and PLD inhibitor treatment. **a** Schematic presentation of the effect of DGK and PLD inhibitors on DAG and PA metabolism. **b** HEp-2 cells were subjected to sulfation assay with ricin-sulf1 (RS1) after treatment with the 10 µM of the indicated inhibitors for 1 h. The upper figure shows an autoradiogram and the western blot against ricin for the same membrane from one representative experiment and the lower figure shows the quantification of RS1 sulfation expressed as percent of control, *n* ≥ 3. **c** RS1 sulfation was measured as described above after treatment with RS1 for the indicated time points. The upper figure shows an autoradiogram from one representative experiment and the lower figure shows the quantification of RS1 sulfation, *n* = 3. **d** RS1 sulfation was measured as described above in CaCo-2, PC-3 and U-2 OS cells, *n* = 3. **e** U-2 OS cells were treated with 10 µM of the indicated inhibitors for 1 h, followed by a 30 min pulse with 4 µg/ml RS1 and a 1 h chase. Cells were fixed, permeabilized, stained with antibodies against ricin (red) and Golgin97 (green) and mounted in ProLong Diamond with DAPI (blue). Left: Representative confocal images; scale bar, 10 µm. Right: To quantify RS1 transport to the Golgi, at least 29 individual cells from 3–6 confocal images for each condition were manually marked and analyzed using Fiji for each experiment. The upper figure shows the mean intensity of ricin in the Golgin97 mask in individual cells from one representative experiment, the lower figure shows the summary of means from three independent experiments

To modulate the DAG and PA levels in the cells, we have used the DGK inhibitors R59022 (RI) and R59949 (RII) and the PLD inhibitors CAY10593 (CAY93) and CAY10594 (CAY94) (Fig. 1a). These inhibitors have been reported to have different selectivities for DGK and PLD isoforms. Both DGK inhibitors strongly inhibit the DGKα isoform; RI moderately inhibits DGKε and DGKθ, whereas RII strongly inhibits DGKγ and moderately inhibits DGKδ and DGKκ [30]. CAY93 primarily inhibits PLD1, whereas CAY94 primarily inhibits PLD2 [31]. To determine the effect of DAG and PA modulation on transport to the Golgi apparatus, we have taken advantage of the sulfation process that occurs specifically in the trans-Golgi network (TGN). Using modified protein toxins containing sulfation sites that can be labeled with radioactive sulfate, we can monitor retrograde transport to the Golgi. Treatment with both DGK and PLD inhibitors strongly increased sulfation of ricin-sulf1 (Fig. 1b) in HEp-2 cells, suggesting that retrograde transport is increased when DAG and PA levels are altered. Combination of the DGK and PLD inhibitors gave an additive effect, indicating that DGK and PLD inhibition may increase transport by different mechanisms. The inhibitors did not affect the sulfation process per se, as total protein sulfation was not affected. Since RI and CAY94 had a tendency for higher effect on ricin sulfation compared to RII and CAY93, respectively, we decided to focus on the DGK inhibitor RI and the PLD inhibitor CAY94 for the rest of the study.

Next, by measuring ricin sulfation at different time points ranging from 30 to 90 min, we investigated how the inhibitors affect the kinetics of the retrograde transport of ricin. We found that ricin was more rapidly transported to the Golgi after both DGK and PLD inhibition, and detectable levels of sulfated toxin could be seen already after 45 min in inhibitor-treated cells, whereas only faint bands were visible after 60 min in control-treated cells (Fig. 1c). Stronger band intensities also indicate that more ricin was able to reach the Golgi after DGK and PLD inhibition. The increase in retrograde transport of ricin is not restricted to HEp-2 cells, as we also saw a similar, although lower, increase in ricin sulfation after DGK and PLD inhibition in Caco-2, PC-3 and U-2 OS cells (Fig. 1d).

To visualize ricin transport to the Golgi, cells were treated with a 30 min ricin-sulf1-pulse and chased for 60 min in the presence of 1 mM lactose to prevent reuptake of recycled toxin. We then looked at ricin colocalization with the TGN marker Golgin-97 by immunofluorescence confocal microscopy. As the Golgi morphology is highly variable in HEp-2 cells, we used U2-OS cells for this assay. After ricin-sulf1 pulse-chase, we could see a clear perinuclear staining partially overlapping with the TGN marker (Fig. 1e), and quantification of the ricin intensity/area in the TGN (Golgin-97 positive structures) showed an increase after inhibitor treatment, in agreement with the sulfation data.

To further support the notion that retrograde transport of ricin is increased after DGK and PLD inhibition, we measured ricin transport to the ER and into the cytosol. ER transport was measured using a modified ricin molecule containing both sulfation and glycosylation sites (ricin-sulf2). N-linked glycosylation occurs in the ER and controls proper folding of proteins, and in the presence of radioactive mannose, ricin-sulf2 will be radioactively labeled when it reaches the ER. As shown in Fig. S1a, ricin mannosylation was increased after treatment with DGK and PLD inhibitors, corroborating the idea that retrograde transport is increased. Since ricin-sulf2 also contains a sulfation site, this molecule can be used to investigate transport from the Golgi to the ER when incubated in the presence of radioactive sulfate. Ricin-sulf2 will then be radioactively labeled in the Golgi and upon reaching the ER, glycosylation will increase the size of ricin-sulf2 which can be visualized by autoradiography. Comparison of the intensity of the two ricin-sulf2 bands shows that DGK and PLD inhibition has no effect on transport between the Golgi and the ER (Fig. S1b). Ricin exerts its toxic action by removing an adenine from the 60S ribosomal subunit, thereby preventing protein synthesis. We found that in the presence of DGK and PLD inhibitors, less ricin is needed to inhibit protein synthesis, resulting in a two-fold sensitization towards ricin (Fig. S1c,d). Overall, these experiments clearly show that DGK and PLD inhibition significantly increase the retrograde transport of ricin to the Golgi, the ER and the cytosol.

**Fig. 2:**
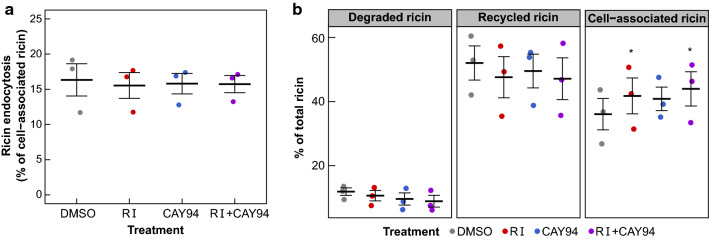
DGK and PLD inhibition do not inhibit the endocytosis, degradation or recycling of ricin. HEp-2 cells were treated with 0.1% DMSO, 10 µM RI and/or 10 µM CAY94 for 1 h followed by incubation with ^125^I-ricin for **a** 20 min to measure ricin endocytosis, *n* = 3 or **b** 20 min plus 2 h chase to measure the amount of ricin degradation and recycling, *n* = 3

The increase in retrograde transport can be caused by a change in endosomal sorting or by increased internalization, and we therefore measured the endocytic uptake after DGK and PLD inhibition using ^125^I-labelled ricin. As shown in Fig. 2a, equal amounts of ricin were internalized, suggesting that DGK and PLD inhibition increases retrograde transport by altering endosomal sorting. We next investigated if sorting into the degradative and recycling pathways was also affected by DGK and PLD inhibition. We treated cells with a 20 min pulse of ^125^I-labelled ricin, followed by a 2 h chase in medium containing lactose to prevent reuptake of ricin and determined the amount of cell-associated, recycled and degraded ricin. There were no significant changes in ricin degradation or recycling after DGK or PLD inhibition (Fig. 2b). Thus, DGK and PLD inhibition seems to increase the endosomal sorting of ricin into the Golgi without affecting the recycling or degradation of the toxin. We have previously shown that transport of ricin to the Golgi apparatus is dependent on Vps34 and formation of PI3P [32]. Thus, we tested whether the Vps34 inhibitor SAR405 could prevent RI- and CAY94-induced increase in ricin transport (Fig. S2). Indeed, SAR405 gave a significant reduction in ricin sulfation when added in combination with RI or CAY94, indicating that the inhibitor-mediated increase in ricin sulfation is dependent on Vps34 activity.

Next, we investigated if the increase in retrograde transport is specific for ricin or if other retrograde cargos are similarly affected. To this end, we studied the Golgi transport of the bacterial toxin Shiga toxin. Shiga toxin binds to the glycosphingolipid Gb3 on the cell surface and is also transported retrogradely from endosomes to the Golgi and the ER [11, 13]. We used a modified Shiga toxin molecule containing sulfation sites (StxB-sulf2) to investigate transport to the Golgi and measured sulfation in a similar manner as described above for ricin. We found that treatment with RI gave a clear increase in StxB-sulf2 sulfation, whereas treatment with CAY94 had a variable effect. In most experiments, CAY94 increased StxB-sulf2 sulfation, but generally had less effect than the DGK inhibitor (Fig. S3a). Colocalization studies between a non-toxic version of Shiga toxin (Stx1m) and the TGN marker Golgin-97 showed a similar trend of increased transport, without reaching statistical significance (Fig. S3b,c). These data suggest that the increase in retrograde transport mediated by DGK and PLD inhibitors is not restricted to ricin, but that not all pathways are changed to the same extent.

### Changes in the lipidome following inhibition of DGK and PLD

**Fig. 3:**
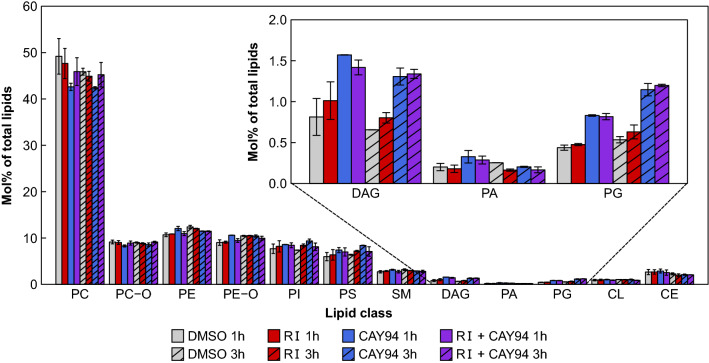
Changes in lipid composition after inhibitor treatment. HEp-2 cells were treated with 0.1% DMSO, 10 µM RI, 10 µM CAY94 or with the combination of the inhibitors for 1 h or 3 h, and whole-cell lysates were analyzed by MS. The figure shows the amount of different lipid classes in the samples expressed as percentage of the total lipid for each sample. The error bars show mean deviation, *n* = 2. For detailed description of the calculations performed on MS data see section “Data analysis and post-processing” in the “Materials and methods” section and the abbreviations of all lipid classes are given under the section “Lipid extraction for MS lipidomics” in the Materials and methods section

**Fig. 4:**
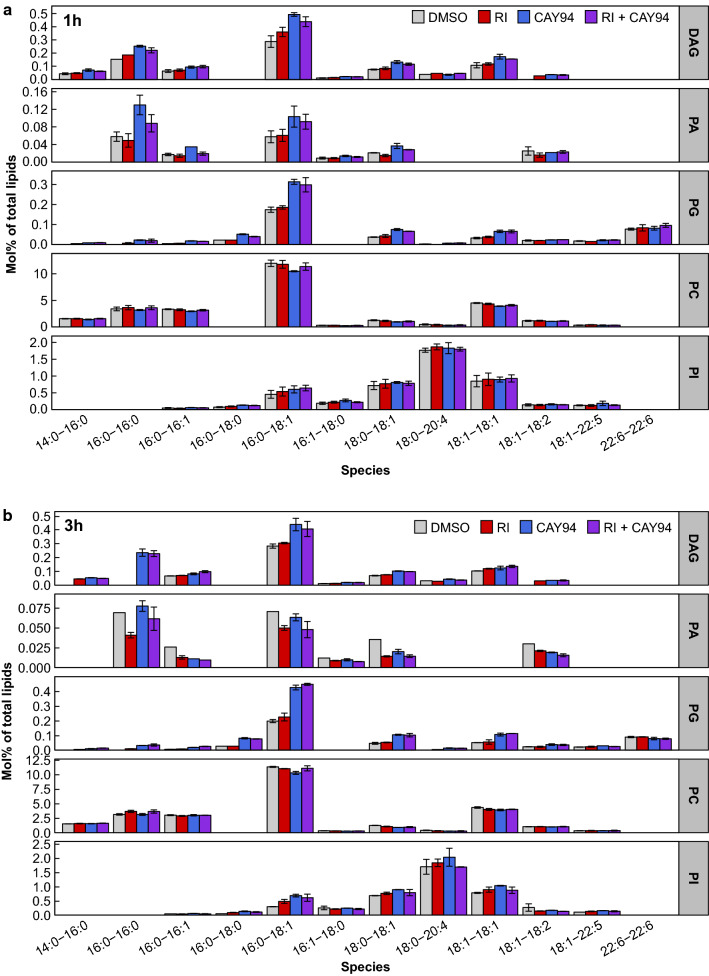
Species composition of DAG, PA, PG, PC and PI after inhibitor treatment. HEp-2 cells were treated with 0.1% DMSO, 10 µM RI, 10 µM CAY94 or with the combination of the inhibitors for 1 h (**a**) or 3 h (**b**), and whole-cell lysates were analyzed by MS. The figure shows relative amount of major DAG, PA and PG species and the equivalent PC and PI species. The error bars show mean deviation, *n* = 2. For detailed description of the calculations performed on MS data see section “Data analysis and post-processing” in the Materials and methods section and the abbreviations of all lipid classes are given under the section “Lipid extraction for MS lipidomics” in the Materials and methods section

Inhibition of DGK is expected to increase DAG levels while decreasing PA levels, whereas PLD inhibition can be expected to decrease PA and perhaps also DAG (Fig. 1a). To test whether this was indeed the case, we treated cells with inhibitors for 1 or 3 h and measured the changes in the cellular lipidome by MS lipidomics. As expected, after both 1 and 3 h, the DGK inhibitor RI gave a weak, but consistent, increase in DAG levels without affecting any of the other lipid classes measured (Fig. 3 and Supplementary material 2). The PLD inhibitor CAY94, however, gave a surprising transient increase in PA that was reversed after 3 h and a persistent increase in DAG and PG (Fig. 3 and Supplementary material 2). The inhibitors increased the levels of several DAG, PA and PG lipid species, with the most prominent effect on 16:0–18:1 (Fig. 4). DAG 16:0–16:0 was also increased after 1 h treatment with the PLD inhibitor and PA was increased after 1 h treatment with the PLD inhibitor. The DAG, PA and PG species that were most affected by inhibitor treatment share the same fatty acyl composition as the most abundant PC species. There were no major changes in the 18:0–20:4 species of DAG, PA and PG, which is the most abundant PI species, suggesting that PC is the source of the increased DAG, PA and PG levels (Fig. 4).

It should be noted that after 3 h of inhibitor treatment, one of the major DAG species, 16:0–16:0, is completely lost from control and RI-treated samples. During inhibitor treatment, cells are serum-starved. Our preliminary data show that incubations in the absence of serum reduced the cellular DAG levels in a time-dependent manner and that 1 h and 3 h in the absence of serum gave similar DAG levels as found in the cells treated with DMSO for 1 and 3 h (Fig. 3 and Supplementary material 2 and 3). Interestingly, treatment with the PLD inhibitor CAY94 gave similar DAG levels as in cells grown in complete medium. The DAG species most affected by removal of serum were 16:0–16:0 and 16:0–18:1 (Supplementary material 3), the same species that were most changed by CAY94 treatment (Fig. 4).

### The effects of different PLD inhibitors on PLD activity and ricin transport

Given the surprising effects of the PLD inhibitor on the cellular PA level, we wanted to verify that the inhibitor actually inhibits PLD activity. To this end, we used a modified version of the recently described IMPACT (Imaging Phospholipase D Activity with Clickable alcohols via Transphosphatidylation) method to measure PLD activity in living cells [33]. Basal PLD activity depends both on PLD1 and PLD2, while phorbol ester PMA stimulates mainly PLD1 [33]. We tested the inhibitor efficiency on both basal and PMA-stimulated PLD activity and found that at a concentration of 10 µM, CAY94 inhibited both the basal and PMA-stimulated PLD activity, indicating that at this concentration, CAY94 inhibits both PLD1 and PLD2 (Fig. S4). Thus, the increase in PA seen in the lipidomics assay is not due to inefficient PLD inhibition. Based on our sulfation data, both CAY93 and CAY94 increased ricin transport (Fig. 1b). The IC50 values of CAY93 in cells are 11 nM for PLD1 and 1.8 µM for PLD2, and for CAY94, the IC50 values are 1 µM for PLD1 and 110 nM for PLD2 [31]. The 10 µM concentration of CAY93 and CAY94 should therefore inhibit both PLD1 and PLD2, while at 1 µM they should be isoform specific. In agreement with the reported IC50 values, 1 µM CAY93 efficiently blocked PMA-induced PLD activity, but had only a slight reduction in basal PLD activity, whereas 1 µM CAY94 efficiently reduced basal PLD activity and had no effect on PMA-induced PLD activity (Fig. S4). At 1 µM concentrations, CAY93 and CAY94 gave only a slight increase (15–40%) in ricin-sulf1 sulfation, suggesting that both PLD1 and PLD2 need to be inhibited to strongly stimulate ricin transport to the Golgi (Fig. S5). To investigate whether PLD1 and PLD2 need to be inhibited, we also tested the dual PLD1 and PLD2 inhibitor FIPI. At 1 µM concentration, FIPI strongly inhibited both basal and PMA-stimulated PLD activity (Fig. S4), in agreement with published IC50 values in a nanomolar range for both PLD1 and PLD2 [31]. In ricin-sulf1 sulfation assays, FIPI gave similar results as CAY93 and CAY94 when used at 1 or 10 µM (Fig. S5). The CAY inhibitors and FIPI inhibits PLD via binding to its HKD domain. We also tested a new PLD inhibitor VU 0364739 (VU036), which in addition to binding to the HKD domain, also interacts with an allosteric site of PLD and has IC50 values 1.5 µM for PLD1 and 20 nM for PLD2 in cells [34]. At 1 µM concentration, VU036 effectively inhibited basal PLD activity but had no effect on PMA-induced PLD activity, while at 10 µM concentration, VU036 blocked both basal and PMA-induced PLD activity (Fig. S6a,b) which is in agreement with the published IC50 values [34]. When tested in the ricin sulfation assay, VU036 gave very similar results as CAY94, with no or very little effect at 1 µM concentration and a high increase in ricin sulfation at 10 µM (Fig. S6c).

### Several PLD and DGK isoforms are involved in regulating ricin transport

Based on The Human Protein Atlas, HeLa cells, which have a similar karyotype as HEp-2 cells [35], express four isoforms of PLD (PLD1, PLD2, PLD3 and PLD6), and seven isoforms of DGK (α, β, δ, ε, ζ, η and θ) [36, 37]. Therefore, we chose to test the expression levels of three PLD isoforms, PLD1, PLD2 and PLD3 (we did not include the mitochondrial PLD6), and six DGK isoforms, α, δ, ε, ζ, η and θ (we did not include β, which is enhanced in brain tissue [38] and has very low expression in HeLa cells [36]). Gene expression was analyzed by qPCR and we found that HEp-2 cells have high expression of PLD3 and lower expression of PLD1 and PLD2 (Fig S7), which is similar to the expression pattern of PLDs in HeLa cells. It should be mentioned that the primer efficiency for PLD2 was lower than for the other primers, leading to higher Cp values and underestimation of the expression level. For the DGK expression in HEp-2 cells, the highest mRNA level was observed for DGKδ (Fig S7), which is similar to HeLa cells. However, the expression of some DGK isoforms seems to differ between HEp-2 and HeLa cells: DGKθ is one of the abundant DGK isoforms in HeLa cells, while it was least expressed of all of the analyzed DGKs in HEp-2 cells (Fig. S7). On the contrary, we found DGKε to be the second most abundant of the six DGK isoforms tested in HEp-2 cells, while in HeLa cells, it has lowest expression of the six [36].

**Fig. 5:**
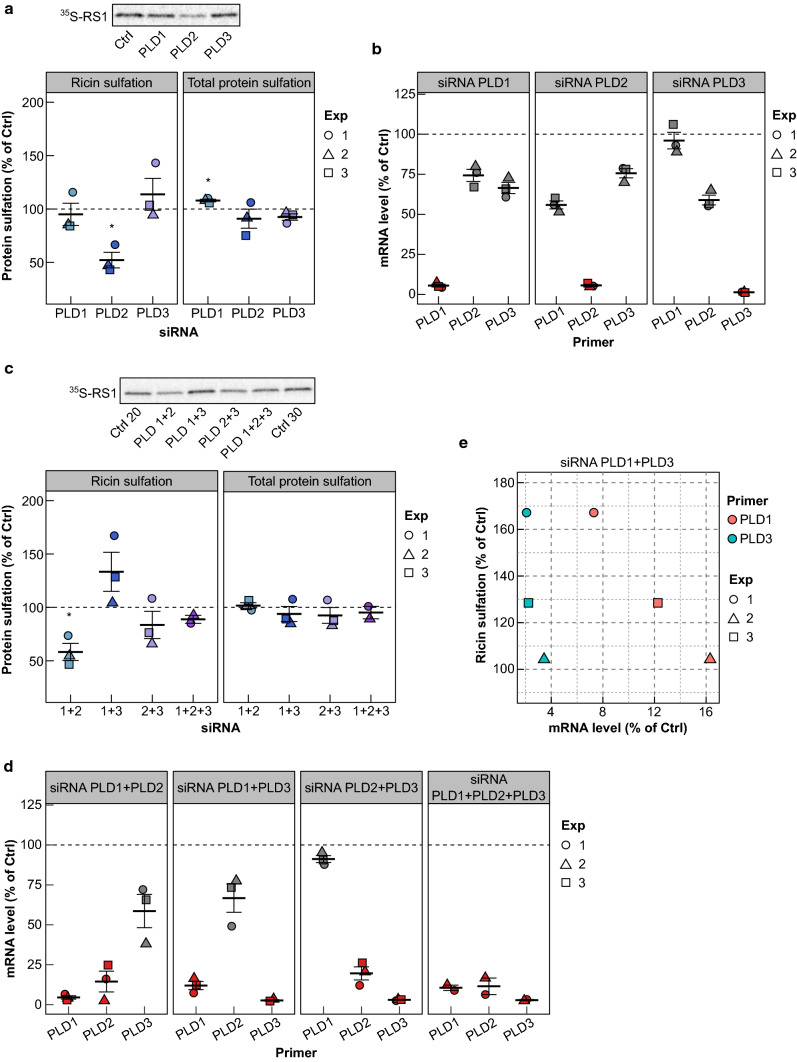
Ricin transport to the Golgi after knockdown of different PLD isoforms. **a**, **b** HEp-2 cells were transfected with 10 nM siRNA against PLD1, PLD2 o PLD3, or with 10 nM control siRNA for 48 h and they were then subjected to sulfation assay with RS1 or analyzed for mRNA levels using qPCR. **a** The upper figure shows an autoradiogram from one representative experiment, and the lower figure shows the quantification of RS1 sulfation and total protein sulfation expressed as percent of control (*n* = 3). **b** mRNA levels of different PLD isoforms normalized to reference gene (TBP) and expressed as percent of control. Red color indicates the isoform which was targeted by the siRNA used in the sample. **c**–**e** HEp-2 cells were transfected with combination of either two siRNAs against PLD1, PLD2 o PLD3 or all three siRNAs (10 nM each), or with 20 nM or 30 nM control siRNA for 48 h and they were then subjected to sulfation assay with RS1 or analyzed for mRNA levels using qPCR. **c** The upper figure shows an autoradiogram from one experiment, and the lower figure shows the quantification of RS1 sulfation and total protein sulfation expressed as percent of control (*n* = 3 for double knockdown and *n* = 2 for triple knockdown). **d** mRNA levels of different PLD isoforms normalized to reference gene (TBP) and expressed as percent of control. Red color indicates the isoforms which were targeted by the siRNAs used in the sample. **e** The figure shows the dependence between mRNA levels for PLD1 and PLD3 and RS1 sulfation in the cells treated with the combination of siRNA against PLD1 and PLD3. As shown, the best knockdown of PLD1 and PLD3 gives the highest RS1 sulfation

Based on the expression pattern of PLD and DGK isoforms in HEp-2 cells, we chose to knock down PLD1, PLD2, PLD3, DGKα, DGKδ, DGKε, DGKζ and DGKη by siRNA for 48 h and then analyzed ricin transport in these cells. Knockdown of PLD1 or PLD3 did not have a significant effect on ricin sulfation, while knockdown of PLD2 led to a significant reduction in the sulfation of ricin (Fig. 5a), without affecting its binding or uptake (Fig S8). The knockdown efficiency was more than 90% for all three siRNAs, and the downregulation of one isoform also affected the mRNA levels of other isoforms but to a lower extent than the targeted isoform (Fig. 5b). Since our inhibitor data indicated that more than one PLD needs to be inhibited to increase ricin transport to the Golgi, we also tested double and triple knockdown of the PLDs. Similar to knockdown of PLD2 only, combined knockdown of PLD1 and PLD2 gave a significant reduction in ricin transport, while combined knockdown of PLD1 and PLD3 led to varying increase in ricin sulfation (Fig. 5c). Importantly, the increase in ricin sulfation after the double knockdown of PLD1 and PLD3 correlates with the knockdown efficiency of PLD1 and PLD3: higher knockdown efficiency of PLD1 and PLD3 led to higher increase in ricin sulfation (Fig. 5d,e), which, together with the lack of effect on ricin sulfation after single knockdown of PLDs, shows that both of these enzymes need to be efficiently knocked down to increase ricin transport to the Golgi.

**Fig. 6:**
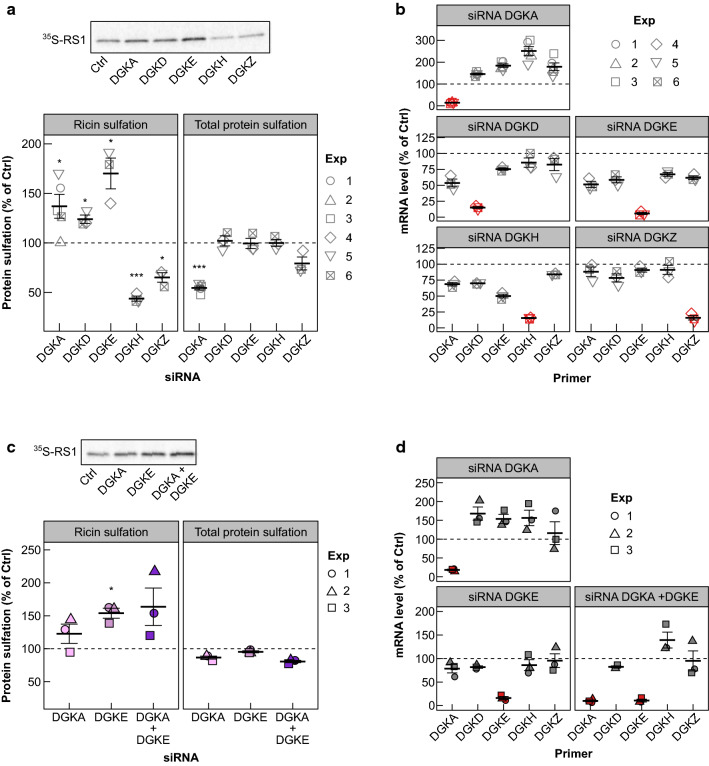
Ricin transport to the Golgi after knockdown of different DGK isoforms. **a**, **b** HEp-2 cells were transfected with 10 nM siRNA against DGKα (DGKA), DGKδ (DGKD), DGKε (DGKE), DGKη (DGKH), DGKζ (DGKZ) or with 10 nM control siRNA for 48 h and they were then subjected to sulfation assay with RS1 or analyzed for mRNA levels using qPCR. **a** The upper figure shows an autoradiogram from one experiment, and the lower figure shows the quantification of RS1 sulfation and total protein sulfation expressed as percent of control (*n* = 3; except for DGKA: *n* = 5). **b** mRNA levels of different DGK isoforms normalized to reference gene (TBP) and expressed as percent of control. Red color indicates the isoform which was targeted by the siRNA used in the sample. **c**–**e** HEp-2 cells were transfected with 10 nM siRNA against DGKα (DGKA), DGKε (DGKE), or the combination of the two. As a control, the cells were transfected with 20 nM control siRNA and 10 nM of control siRNA was also added to single treatment samples to have 20 nM siRNA in all wells. After 48 h, the cells were subjected to sulfation assay with RS1 or analyzed for mRNA levels using qPCR. **c** The upper figure shows an autoradiogram from one experiment, and the lower figure shows the quantification of RS1 sulfation and total protein sulfation expressed as percent of control (*n* = 3). **d** mRNA levels of different DGK isoforms normalized to reference gene (TBP) and expressed as percent of control. Red color indicates the isoforms which were targeted by the siRNAs used in the sample

The single knockdown of all tested DGK isoforms had a significant effect on ricin sulfation: the knockdown of DGKα, DGKδ and DGKε gave an increase in ricin sulfation, while the knockdown of DGKζ and DGKη led to a reduction in ricin sulfation (Fig. 6a). The knockdown of DGKα also gave a significant reduction in total protein sulfation (Fig. 6a) and also led to increased expression of all other isoforms of the DGKs (Fig. 6b), making it difficult to say whether DGKα has a direct effect on ricin transport. Since DGKα and DGKε are the main targeted isoforms by RI [30], we also tested whether combined knockdown of the two could lead to an even higher increase in ricin transport to the Golgi. However, the combined knockdown of DGKα and DGKε did not give significantly higher increase in ricin sulfation than the knockdown of DGKε alone (Fig. 6c).

### The DAG effectors PKC and PKD are not involved in upregulating retrograde transport after DGK and PLD inhibition

**Fig. 7:**
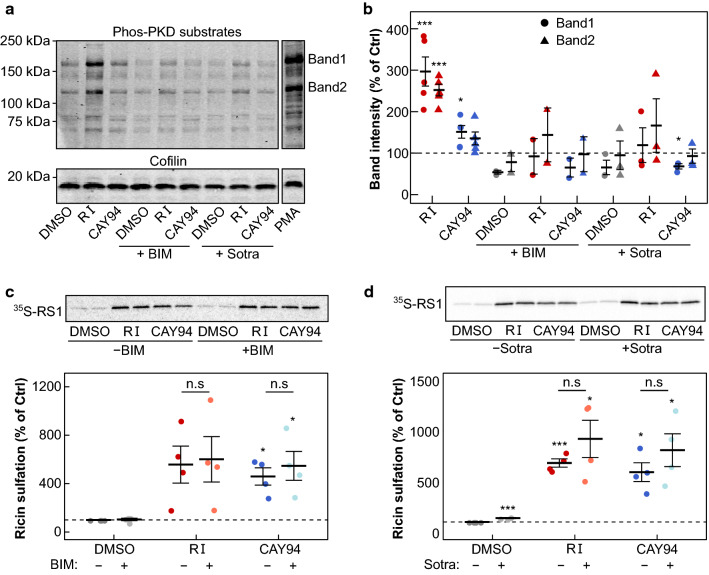
The RI- and CAY94-mediated increase in ricin transport is not dependent on the DAG substrate PKC. **a** The phosphorylation of PKD substrates was measured by Western blot after treating HEp-2 cells with 10 µM RI or 10 µM CAY94 in the presence or absence of the PKC inhibitors BIM I (BIM; 10 µM) and Sotrastaurin (Sotra; 1 µM) for 1 h. Cofilin was used as a loading control. One representative experiment is shown. **b**, **c** HEp-2 cells were subjected to sulfation assay with ricin-sulf1 (RS1) after treatment with 10 µM RI or 10 µM CAY94 in the presence or absence of the PKC inhibitors **b** BIM (10 µM) and **c** Sotra (1 µM) for 1 h. The upper figures show representative autoradiograms and the lower figures show quantifications of RS1 sulfation expressed as percent of control (*n* = 4)

Both the DGK and the PLD inhibitor resulted in increased DAG levels, and thus we next investigated whether membrane recruitment of DAG-binding proteins could play a role in increasing sorting into the retrograde pathway. PKC is the most characterized DAG-binding protein and it has previously been shown that downregulation of PKCδ strongly reduced the retrograde transport of Shiga toxin [39]. PKD, another DAG-binding protein, has been shown to be essential in the secretory pathway, where it activates PI4K in the Golgi apparatus and contributes to the generation of cell surface specific transport carriers [2, 40]. Activation of PKD was tested by Western blotting using an antibody that detects proteins containing phosphorylated Ser/Thr at the PKD consensus sequence. Treatment with the DGK inhibitor gave an increase in phosphorylated proteins (Fig. 7a), suggesting an increase in PKD membrane translocation and activity, in agreement with the increase in DAG seen in the lipidomics assay. Although treatment with the PLD inhibitor gave a stronger relative increase in DAG levels, it gave only a small increase in phosphorylated PKD substrates (Fig. 7a). PKC can act as an upstream activator of PKD [41] and treatment with the PKC inhibitors bisindolylmaleimide I (BIM) and sotrastaurin (Sotra) strongly inhibited the RI-induced phosphorylation of PKD substrates (Fig. 7a). To check whether PKC activity was important for the increased retrograde transport of ricin after DGK or PLD inhibition, we measured ricin-sulf1 sulfation after treatment with RI or CAY94 in the presence or absence of PKC inhibitors. The PKC inhibitors did not affect the increase in ricin-sulf1 sulfation after DGK or PLD inhibition (Fig. 7b,c), indicating that it is not caused by increased PKC activity. Since the PKC inhibitors also prevented RI-mediated PKD activation, we reason that the DAG effectors PKC and PKD are dispensable for the up-regulation of ricin-sulf1-sulfation after DGK- and PLD inhibition.

### DGK and PLD inhibitors alter the morphology of endosomes

**Fig. 8:**
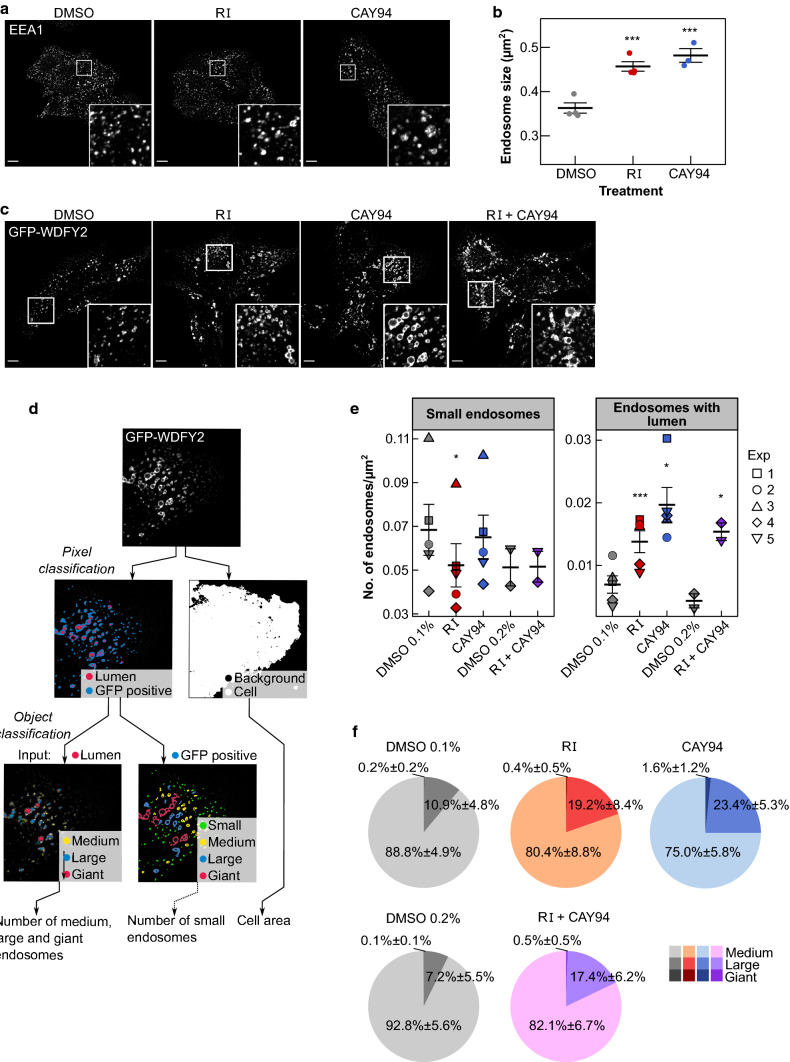
Treatment with DGK and PLD inhibitors alters endosome morphology. **a**, **b** HEp-2 cells were treated with 10 µM RI or 10 µM CAY94 for 1 h and stained with antibodies against EEA1. **a** Representative images of EEA1-positive endosomes in fixed cells from confocal microscopy; scale bar, 10 µm. **b** Quantification of endosome size from three independent experiments. **c**–**f** RPE GFP-WDFY2 cells were treated with 10 µM RI, 10 µM CAY94 or with a combination of 10 µM RI and 10 µM CAY94 for 1 h. **c** Representative images of GFP-WDFY2 positive endosomes in fixed cells from confocal microscopy; scale bar, 10 µm. **d** The pipeline used for image analysis. **e** The number of small endosomes (classified as small in the object classification of GFP-positive pixels) and the number of endosomes with detected lumen (total number of objects in the object classification of luminal pixels) per cell area. **f** Distribution of medium, large and giant endosomes within the subpopulation of endosomes with detected lumen; mean values ± STD, *n* = 5 for DMSO 0.1%, RI and CAY94, *n* = 2 for DMSO 0.2% and RI + CAY94

By altering the DAG levels, the inhibitors may also change the biophysical properties of the membrane, and a local increase in the concentration of DAG has been shown to affect both fission- and fusion processes [2, 42]. We therefore studied how treatment with the DGK and PLD inhibitors affect endosomal morphology. HEp-2 cells were treated with inhibitors and stained with antibodies against the endosomal marker EEA1 and investigated by immunofluorescence confocal microscopy. Both RI and CAY94 gave a significant increase in the size of EEA1-positive structures, and we also noticed a more irregular shape of the endosomes (Fig. 8a,b).

To investigate whether inhibitors affect biophysical properties of endosomal membranes, we used an environment-sensitive probe NR12S, which has been proven useful in analyzing plasma membrane order in living cells [43, 44]. Recently, the NR12S probe has also been employed to probe the membrane packing in the endocytic recycling compartment, as it is taken up by non-selective endocytosis and delivered to the endocytic recycling compartment [45]. Thus we employed NR12S to analyze whether PLD or DGK inhibition affects membrane packing in the endosomes. HEp-2 cells were incubated with Alexa647-labeled transferrin to mark early and recycling endosomes and then stained with the NR12S probe and imaged using a Zeiss LSM 780 confocal microscope (Fig. S9a). The mean generalized polarization (GP) value was quantified within the plasma membrane and endosome (transferrin-positive) mask as described in the Supplementary material 1. The GP value in the transferrin-positive endosomes was higher than the GP value in the plasma membrane (Fig. S9b), indicating higher lipid packing in the endosome membrane, which is in agreement with results in [45]. Since the NR12S probe has a slow flipping rate across the bilayer [43], it should mainly be localized at the outer leaflet of the plasma membrane and the luminal leaflet of the endosomes in the time frame of the experiment. As shown, inhibitor treatment did not affect the GP value at the plasma membrane (at least at the outer leaflet, which is probed by NR12S), but there seemed to be an increase in the GP value at the endosomes after PLD inhibition (Fig. S9b) possibly mediated via interaction of the two leaflets of the membrane and/or flipping of lipids, such as DAG, from the cytosolic to the luminal side of the endosomal membrane. The absolute value of GP depends on the imaging settings and cannot be compared between individual experiments without calibration; therefore we calculated the difference between the GP value at the endosomes and the GP value at the plasma membrane for each cell (delta GP). The deltaGP values represent the difference in membrane packing between the plasma membrane and the endosomes, and can be used to combine data from individual experiments. Using this approach, we saw a significant increase in deltaGP in the PLD inhibitor treated cells, but no significant change after DGK inhibitor treatment (Fig. S9c).

To study the endosomal morphology in more detail, we used an RPE1 cell line stably expressing GFP-WDFY2. WDFY2, which interacts with VAMP3, was recently described as an endosomal protein localizing to endosomal tubules and found to regulate endosomal sorting [22]. Under conditions of mild overexpression, WDFY2 also labels the limiting membrane of the endosome, allowing us to study endosomal morphology. After treatment with RI and CAY94, we saw an increase in endosome size and clustering (Fig. 8c). To quantify this increase, Ilastik software [24] was used for automated pixel and object classification, allowing us to quantify the number of small, medium, large/clustered and giant/clustered endosomes, as shown in Fig. 8d. The number of small endosomes was similar in control- and CAY94-treated cells, but was reduced after RI treatment (Fig. 8e), whereas the number of endosomes with visible lumens was strongly increased after both RI and CAY94 treatment (Fig. 8e). The distribution of medium, large and giant endosomes within the subpopulation of endosomes with visible lumens was also changed after RI and CAY94 treatment. CAY94-treatment gave a larger proportion of giant endosomes than RI and control treatment, whereas both RI and CAY94-treatment increased the proportion of large endosomes (Fig. 8f). Interestingly, cells treated with the combination of RI and CAY94 seem to have the combination of the morphological changes induced by the inhibitors individually: many large endosomes that are more clustered than in cells treatment with CAY94 alone (Fig. 8c).

To test whether there is a possible link between changes in the endosome morphology and the increase in ricin transport, we analyzed WDFY2-positive endosome size in the cells treated with two different concentrations (1 µM and 10 µM) of CAY94 and FIPI. As shown in Fig. S10, at 1 µM concentration, PLD inhibitors did neither affect the number of endosomes with detected lumen, nor the endosomes size distribution, while at 10 µM concentration, both CAY94 and FIPI gave a clear increase in the number of endosomes with lumen as well as an increase in their size. It seems that the changes in the endosomal size correlate with the increase in ricin sulfation, as 10 µM is required to give a strong increase in the ricin transport (Fig. S10).

**Fig. 9:**
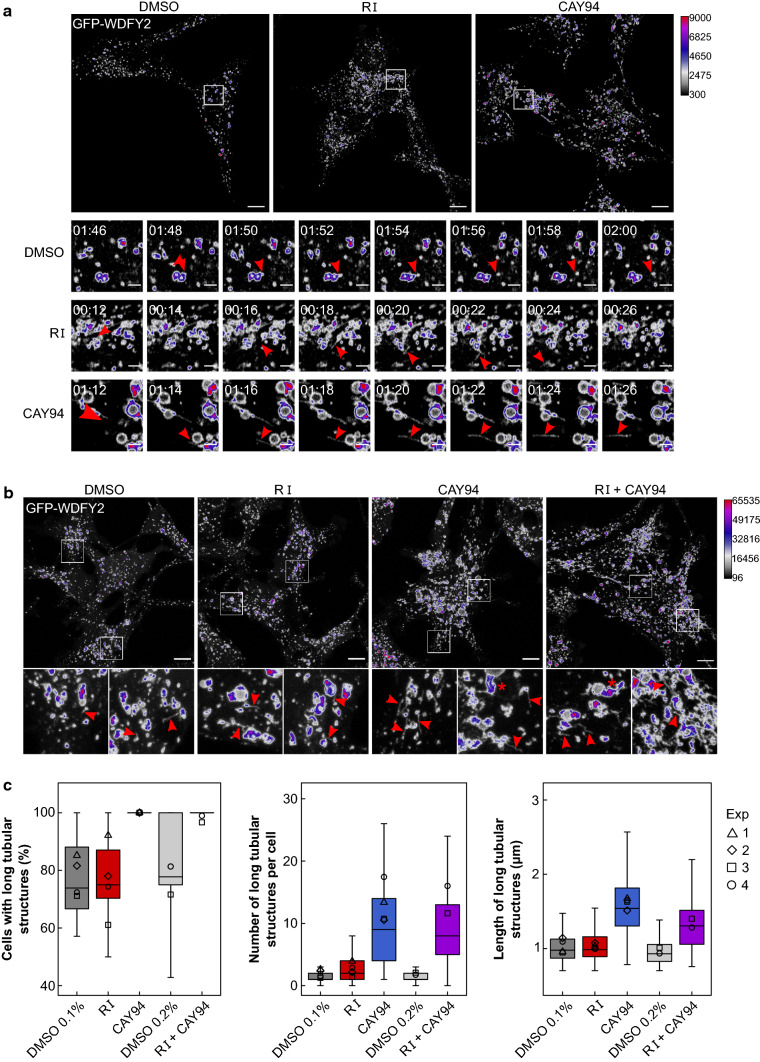
Treatment with DGK and PLD inhibitors increases endosome tubulation. **a** RPE GFP-WDFY2 cells were treated with 10 µM RI or 10 µM CAY94 for 1 h and imaged each 2 s for 2 min. Example still images are shown with zoomed-in time series underneath; the arrow heads indicate forming WDFY2-positive tubules; scale bars, 10 µm (main images) and 2 µm (zoomed-in images). Example videos are given in Supplementary file 4. **b**, **c** RPE GFP-WDFY2 cells were treated with 10 µM RI, 10 µM CAY94 or with a combination of 10 µM RI and 10 µM CAY94 for 1 h, fixed, mounted and imaged using 3D scanning. **b** Representative maximum intensity projections from the 3D scans are shown with zoomed-in example images; the arrow heads indicate WDFY2-positive tubules; the star (*) indicates clustered tubules (thick WDFY-2 positive tubules) characteristic for the CAY94 treated cells. **c** All visible tubules were measured and classified as long if were of at least 0.7 µm. The figures show the percentage of cells with visible long tubules, the number of long tubules per cell and the mean length of the long tubules. The empty shapes show the means for each independent experiment (*n* = 4 for DMSO 0.1%, RI and CAY, *n* = 2 for DMSO 0.2% and RI + CAY94); the box plots show pulled data of all independent experiments (at least 25 cells were analyzed for each condition in each experiment)

Retrograde cargo is sorted towards the Golgi from tubular endosomal structures, therefore we also investigated whether the inhibitors increased endosomal tubulation. To this end, we looked at tubule dynamics by live-cell imaging of RPE GFP-WDFY2 cells. Tubulation events were more frequent in RI and CAY94-treated cells than in control-treated cells and we could observe long tubular carriers being released from endosomes travelling through the cytosol (Fig. 9a and Supplementary material 4). For quantification, we fixed cells and performed 3D scans of the cells to capture all visible tubules (Fig. 9b). First, we determined the proportion of cells with visible tubular structures by manual inspection. After CAY94 treatment, all cells were positive for tubular structures, and these cells also had a higher number of long tubular structures (> 0.7 µm) and a higher mean length of the tubules, while treatment with RI increased only the number of long tubular structures per cell without affecting the mean length (Fig. 9c). The combined treatment of RI and CAY94 resembled treatment with CAY94 alone, but induced more clustered endosomes and thus slightly shorter tubules (Fig. 9c). Treatment with DGK and PLD inhibitors increases endosome size and tubulation, but to a different extent and lead to different morphology, indicating that these inhibitors affect the endosome morphology via different pathways.

**Fig. 10:**
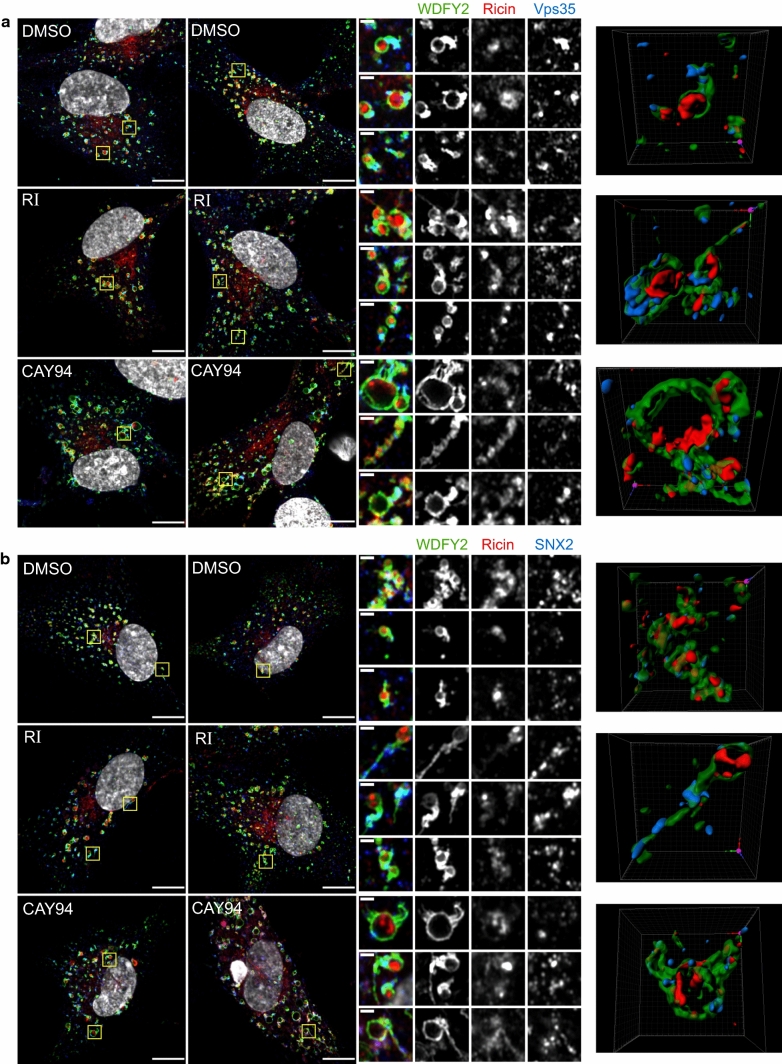
Ricin is in WDFY2-positive structures in close proximity to Vps35 and SNX2. RPE GFP-WDFY2 cells were treated with 10 µM RI or 10 µM CAY94 for 1 h followed by 20 min pulse with 1 µg/ml ricin-Alexa555. Then, lactose was added (final conc. 0.1 M) to remove surface bound ricin-Alexa555 and the cells were fixed, permeabilized, stained with antibodies against **a** Vps35 or **b** SNX2 and mounted in ProLong Diamond with DAPI. High resolution 3D images were acquired using Zeiss LMS 880 Airyscan microscope. Left: Representative 2D images after super-resolution processing; scale bar, 10 µm. Middle: Zoom in on endosomal structures; scale bar, 1 µm. Right: 3D visualization of the endosomal structures shown in the first row for each condition. Colors: green: WDFY2, red: ricin-Alexa555, blue: Vps35/SNX2, white: DAPI

Retrograde cargo destined to the Golgi can be segregated into tubular structures by retromer and the SNX-BAR complexes [46]. To analyze whether ricin is transported via WDFY2-positive endosomes and/or tubules, we treated RPE-GFP-WDFY2 cells with the inhibitors and then added ricin-Alexa555 prior to cell fixation. The cells were immunolabeled for the retromer component Vps35 and the SNX-BAR component SNX2 and imaged using fluorescence super-resolution imaging. Indeed, we clearly saw ricin inside the WDFY2-positive endosomes in control and in inhibitor treated cells. In addition, some of the WDFY2-positive tubules were also positive for ricin, although the signal for ricin was always strongest within the endosomal lumen (Fig. 10). Interestingly, CAY94 treatment often led to endosomes with thick tubular tails positive for WDFY2. Based on the 3D reconstruction, these structures look as multiple tubular structures extending from the endosome. Worth noticing, ricin was often accumulated at the base of such thick tubular structures (Fig. 10). Finally, both Vps35 and SNX2 decorated WDFY2-positive endosomes and tubules and they were often found located at the same position or close to the ricin signal within the endosome (Fig. 10).

## Discussion

One important finding in the present study is that retrograde transport from endosomes to the Golgi apparatus of the protein toxin ricin can be strongly upregulated in HEp-2 cells by affecting lipid composition with inhibitors of DGK and PLD. A similar regulation of ricin transport was seen also in other cell lines, demonstrating that this is a general phenomenon. DGK depletion showed that different DGK isoforms affect ricin transport differently. Knockdown of the *α*, *δ* and *ε* isoforms increased retrograde ricin transport, whereas *η* and *ζ* knockdown led to a reduction. This is line with the results obtained with the DGK inhibitors, which mainly targets the *α* isoform. The data obtained with the different PLD inhibitors together with the siRNA knockdown of different PLD isoforms indicate that it is not sufficient to inhibit a single PLD isoform to get the increase in ricin transport. Like PLD1 and PLD2, PLD3 also has an HKD domain [8] and we speculate that it could potentially be targeted by all of the PLD inhibitors used. It is not known yet whether PLD3 has enzymatic activity, but data based on siRNA knockdown of PLD3 indicate that it plays a role in intracellular sorting and might have enzymatic activity [9]. However, the IC50 value for PLD3 might be higher than PLD1 or PLD2, thus requiring a higher inhibitor concentration.

The combination of inhibitors of DGK and PLD had an even stronger effect than either one added alone, suggesting that different mechanisms are regulated by interfering with these lipid processing enzymes. The transport to the Golgi was monitored both by sulfation of genetically modified ricin and by immunofluorescence microscopy, and as expected the increase in retrograde transport was associated with an increased toxicity. Remarkably, the transport from the TGN to the ER did not seem to be affected by the inhibitors as the fraction of sulfated ricin that was mannosylated in the ER was unchanged. Since endocytosis, recycling and degradation of ricin were essentially unaffected, it seems that there is a selective effect on ricin transport between endosomes and the Golgi apparatus. We also investigated whether we could see a similar regulation on Golgi transport of Shiga toxin which binds to the glycosphingolipid Gb3, and interfering with DGK did have a significant effect on Golgi transport of Shiga toxin (about twofold), whereas there was essentially no change in transport after PLD inhibition. Thus, there is clearly selectivity in which pathway(s) that are changed. We have here chosen to focus on ricin transport not only because of the large change upon treatment with the different inhibitors, but also since ricin, in contrast to Shiga toxin, does not seem to induce signaling in cells [47]. Shiga toxin, which crosslinks glycolipids at the cell surface, is able to induce signaling that in itself can change intracellular sorting [47].

There are a number of pathways leading from endosomes to the Golgi apparatus, and several factors involved in sorting have been characterized [48, 49]. For many years there has been a discussion about whether Golgi transport has to go via recycling endosomes or whether it can occur by direct transport from early endosomes, and to which extent this might be cargo- and cell type- dependent [50]. It has previously been reported that two proteins localized to the tubular recycling endosomes, EHD1 and EHD3, are involved in Shiga transport to the Golgi. These proteins interact with the PA-binding protein MICAL-L1, and it has been shown that both PLD inhibitors and knockdown of DGKα destroy these structures [50, 51]. The strong increase seen in Golgi transport of ricin after treatment with DGK and PLD inhibitors suggests that ricin does not depend on recycling endosomes for retrograde transport, and supports the idea that there is a regulation of transport from early endosomes [52].

The lipid PI3P, which can be formed by the action of the kinase Vps34, is central for recruitment of several proteins to endosomes, including sorting nexins, such as SNX2 and SNX4. Transport of ricin to the Golgi is reduced after inhibition of Vps34 with inhibitors such as wortmannin and LY294002, by expression of Vps34 mutants and after knockdown of SNX2 and SNX4 [32], but it should be noted that in all these cases retrograde transport is not completely inhibited, a finding which may be due to the multiple pathways which ricin can utilize. Importantly, the upregulated transport of ricin after incubation with RI and CAY94 is to a large extent also inhibited by blocking PI3 kinase, indicating that PI3P is involved also in facilitating the strong increase in toxin going to the Golgi.

Since PI3 kinase is important also for upregulated Golgi transport, we decided to study cells which express fluorescently labeled WDFY2, a PI3P binding protein that was recently shown to restrain matrix metalloproteinase secretion in RPE1 cells and to be enriched in actin-stabilized endosomal tubules. Such tubules are certainly candidates for mediating Golgi transport, since WDFY2 was also shown to interact with VAMP3 [22], a v-SNARE that has been implicated in Shiga toxin transport to the Golgi [53]. By microscopy we could show that the inhibitors of DGK and PLD may affect both endosome size and tubulation, and ricin was found to be present in endosomal tubules with Vps35, a component of the retromer, as well as with SNX2, which is involved in Golgi transport of ricin [32].

It has been suggested that actin-stabilization of tubules is required for efficient retrieval of slow-diffusing cargo from endosomes by allowing sufficient time for cargo accumulation in the tubules before they are pinched off [54]. We observed more and longer tubules after inhibitor treatments, especially after PLD inhibition, which may give ricin more time to enter the tubules. Interestingly, it has been shown that cargo destined for the Golgi apparatus accumulate in retromer-positive subdomains of the early endosomes, whereas recycling cargo labeled the entire limiting membrane. Enrichment to these domains did not affect cargo itinerary, but instead increased the rate of endosome exit and subsequent delivery to the appropriate destination [55]. We observed strong ricin staining at the base of WDFY2 tubules, which is in agreement with a large fraction of ricin being recycled. Ricin was also seen in the WDFY2-positive tubules, and since DGK and PLD inhibition increased the number of tubules, more ricin is likely to associate with retromer endosome subdomains, which may improve transport kinetics. It has been published that ligands destined for different locations can exit the endosome in the same tubule [55], and we cannot exclude that sorting at a later stage than tubule formation is also affected. In addition, the detailed mechanism for scission of tubules destined for the Golgi is still unknown and alterations in lipid composition may change scission kinetics and have different impact on Golgi-retrieval pathways.

To investigate whether the inhibitors of DGK and PLD actually had an effect on the lipid composition of the cells, we analyzed the lipidome after similar incubations as when we studied toxin transport. It should be noted that our lipidomic studies both in earlier articles and as here shown, reveal that one cannot necessarily predict the changes that occur in cells upon a given treatment. We have previously by performing lipidomics discovered that lipid synthesis is regulated in ways that have still not been characterized. For instance, by adding a precursor to plasmalogens, the cells responded by also changing synthesis of glycosphingolipids [56]. The data obtained here show that whereas the DGK inhibitor RI as expected gave a small increase in DAG levels without affecting any of the other lipid classes measured, surprising effects on the lipidome were obtained when using the PLD inhibitor CAY94: DAG was strongly increased after CAY94 treatment, and the PLD product PA, as well as PG, were also increased. DAG, PA and PG all had a similar lipid species distribution as PC, suggesting that PC is more likely than PI to be the source of these lipids. Importantly, both inhibitors increased the levels of DAG, which has a conical shape and thus might promote tubulation. The reason for the observed increase in PA after the treatment with CAY94 is not known, but we speculate that it is important for the cell to keep PA as constant as possible and that the cells compensate by increasing the activity of PC-PLC, thus generating more DAG which in turn in transferred to PA. An increase from 0.81 to 1.57% DAG is associated with an increase in PA from 0.20 to 0.33% during the first hour, but the PA level is normalized within the next two hours. PG increases from 0.44 to 0.83% during the first hour and increases further to 1.15% during the next two hours. PG is synthesized from PA via CDP-DAG (CDP: cytidine diphosphate) to PGP (phosphorylated PG) to PG [7]. Thus, we speculate that the cell compensates for the inhibition of PLD by making PA via DAG and then transfers the extra PA formed to make PG to normalize the level of PA after 3 h. The combination of CAY94 and RI gives similar increase in PA as CAY94 alone, however, the speculations about the effects of CAY94 above may still be correct since RI is not targeting all DGK isoforms [30]. If DGK inhibition is not complete, it seems likely that one can obtain data such as those described above, i.e. that an increase from 0.75 to 1.5% DAG can give an increase of PA from 0.15 to 0.30%. To test whether the increase in DAG, PA and PG upon CAY94 treatment comes from PC, one should specifically block PC-PLC activity in combination with CAY94. Although the PC-PLC activity has been known for years, the mammalian PC-PLC has not been purified and the gene sequence has not yet been resolved [57]. The only commercially available pharmacological inhibitor of PC-PLC (D609) has several off-target effects [58], including inhibition of sphingomyelin synthase at similar concentrations as used to inhibit PC-PLC [59], thus at this point, we are not able to test our hypothesis. It should be noted that a challenge of using lipidomics to study endosomal sorting is that we get the sum of lipids in all cellular compartments. Lipids are sorted along the endosomal pathway and the lipid composition in different organelles may be different from the overall cellular composition. Lipid-modulating enzymes may also have subcellular specificity, and local changes in the lipid composition of endosomes may not be detectable at the cellular level.

As discussed above, one role of the various membrane lipids is to recruit proteins, and there may be a change in membrane association of sorting nexins, the WASH complex and actin. We have previously described how inhibition of glycosphingolipid synthesis may change the localization of SNX1 and SNX2 [60]. However, also the lipids themselves have been shown to be important for fusion; that is lipids that are prone to non-bilayer structure such as DAG [61]. One common effect of RI and CAY94 treatment is that there is an increase in DAG 16:0–18:1. However, with the limited knowledge about the role of different lipid species in cells, more lipidomics has to be included in future studies to understand the complexity of membrane lipids in cell physiology.

## Electronic supplementary material

Below is the link to the electronic supplementary material.

Supplementary file1 (PDF 2185 kb)

Supplementary file2 (XLSX 377 kb)

Supplementary file3 (XLSX 121 kb)

Supplementary file4 (AVI 8833 kb)
