# Supplementary material for: Diacylglycerol kinase and phospholipase D inhibitors alter the cellular lipidome and endosomal sorting towards the Golgi apparatus
Source: Cell Mol Life Sci. 2020 May 23;78(3):985–1009. doi: 10.1007/s00018-020-03551-6 (PMC7897626; doi:10.1007/s00018-020-03551-6)
Supplement: Supplementary file 1 — Supplementary file1 (PDF 2185 kb) [file 18_2020_3551_MOESM1_ESM.pdf]

## Supplementary material 1

# Diacylglycerol kinase and phospholipase D inhibitors alter the cellular lipidome and endosomal sorting towards the Golgi apparatus

Anne Berit Dyve Lingelem[1,2], Simona Kavaliauskiene[1], Ruth Halsne[1,2], Tove Irene Klokk[1,3], Michal A. Surma[4], Christian Klose[4], Tore Skotland[1], Kirsten Sandvig[1,5,*]

1. Department of Molecular Cell Biology, Institute for Cancer Research, the Norwegian Radium Hospital, Oslo University Hospital, Oslo, Norway
2. Current address: Department of Forensic Biology, Oslo University Hospital, Oslo, Norway
3. Current address: Regional Committees for Medical and Health Research Ethics, University of Oslo, Oslo, Norway
4. Lipotype GmbH, Dresden, Germany
5. Department of Biosciences, University of Oslo, Oslo, Norway

Corresponding author: Prof. Kirsten Sandvig. E-mail: ksandvig@radium.uio.no, Telephone: +47 22 78 18 28

# Supplementary methods

## Mannosylation

HEp-2 cells were seeded in 6 well plates at a concentration of $2 \times 10^5$ cells/well one day prior to experiments. Cells were washed twice with glucose-free medium (11966-025, Gibco) supplemented with 1 mM glucose and incubated with 0.1 mCi/ml [$^3$H]-mannose in the same medium for 2 h at 37 °C. Inhibitors were added and the incubation continued for 1 h. Then, 23-26 µg/ml ricin-sulf2 was added for 2 h in the presence of 1 µg/ml of the *N*-glycosylation processing inhibitor swainsonine. The cells were washed twice with 0.1 M lactose in HEPES-buffered medium for 5 min at 37 °C to remove surface-bound ricin-sulf2, then washed with ice-cold PBS and lysed in lysis buffer. Lysates were cleared by centrifugation and ricin-sulf2 was immunoprecipitated overnight at 4 °C from cleared lysates using Protein A sepharose beads with the ricin antibody adsorbed. The immunoprecipitate was washed twice with 0.35% Triton X-100 in PBS, resuspended in Laemmli sample buffer with 100 mM DTT and boiled for 5 min. The immunoprecipitate was separated by SDS-PAGE, blotted onto a PVDF membrane and visualized by autoradiography using Kodak BioMax MS films (Sigma-Aldrich) with intensifier screen. Band intensities were quantified using the Quantity One 1-D Analysis Software (Bio-Rad Laboratories Inc). To determine the total amount of protein mannosylation, proteins from the supernatants after immunoprecipitation were precipitated with 5% TCA (100807, Merck). The TCA precipitate was dissolved in 0.1 M KOH and measured by liquid scintillation counting using a Tri-Carb 2100TR Liquid Scintillation Analyzer (Packard).

## Ricin toxicity

HEp-2 cells were seeded at a concentration of $5 \times 10^4$ cells/well in a 24 well plate one day prior to the experiment. The cells were washed once with leucine-free medium (MEM without leucine (Invitrogen) supplemented with 20 mM HEPES, 2 mM GlutaMAX (Thermo Fisher Scientific) and 100 U/ml Penicillin and 100 µg/ml Streptomycin, pH 7.7) before being incubated with inhibitors in leucine-free medium for 1 h at 37 °C. Subsequently, increasing concentrations of ricin holotoxin was added and the incubation continued for 3 h. Then, the medium was replaced with 1 µCi/ml [$^3$H]-leucine in leucine-free medium and incubated for 20 min at 37 °C. Proteins were precipitated with 5% TCA, the precipitate was dissolved in 0.1 M KOH and measured by liquid scintillation counting. Ricin toxicity was determined by comparing the concentration of ricin needed to reduce protein synthesis by 50% after treatment with the different inhibitors.

## PLD activity by IMPACT

The method for PLD activity measurement in live cells was adapted from [1]. HEp-2 cells were seeded in 12 well plates. Next day, the cells were washed once with sulfate-free medium and incubated with indicated inhibitors prepared in sulfate-free medium for 1 hour at 37 °C and 5% $CO_2$. For basal PLD activity, 3-azido-1-propanol (AzProp; 776130, Sigma-Aldrich) was diluted in S-free medium and added directly to the cell medium with the final concentration of 0.5 µM, and the incubation continued for 2 h. For stimulated PLD activity, the cells were incubated with 0.5 µM AzProp for 30 min and then PMA was added directly to the medium to a final concentration of 0.1 µM, and the incubation was continued for 30 min. Then, the cells were washed 3 times for 5 min with HEPES-buffered medium at 37 °C prior to incubation with 0.5 µM BDP FL DBCO (cycloalkyne dye for copper-free click-chemistry; A14F0, Lumiprobe GmbH) prepared in HEPES-buffered medium for 10 min at 37 ºC. The cells were then washed with HEPES buffered medium once and detached by Accutase, spun down at 300 g for 5

min and resuspended in ice-cold PBS. Ice-cold 16% formaldehyde solution was added directly to the cell solution to reach final concentration of 4% formaldehyde, the tube was inverted several times to mix and left on ice for 20 min. The cells were then spun at 400 g for 5 min, and the pellet was resuspended in PBS supplemented with 0.05% saponin and 1% BSA (washing buffer). To remove unreacted BDP FL DBCO dye, the cells were washed 3 times with the washing solution: 20 min at room temperature followed by overnight wash at 4 ºC and the final wash for 20 min at room temperature. The tubes with the cells in the washing solution were rotated during the wash to ensure mixing. Following the final washing step, the cells were spun down and the pellet was rinsed with PBS without mixing, then spun down again and resuspended in PBS. The cell solution was then analyzed using a BD LSR II flow cytometer (BD Biosciences). Side and forward scattering were registered and used to gate out cellular debris and cell clusters, and 20 000 cells were measured for each condition. The FITC detection channel was used to register the BDP FL DBCO signal. To quantify the percentage of the cells with high PLD activity, a manual gate was set to get 10 % of the cells with highest BDP FL DBCO signal in the sample treated with 0.1 % DMSO. The percentage of the cells in this gate was then quantified for all other conditions. The gating and quantification were performed using Cytobank [2].

## Generalized polarization (GP) imaging using confocal microscope

HEp-2 cells were seeded in 4-well Mattek coverglass dishes at a density of $6 \times 10^4$ cells/well one day before the experiment. The medium was changed to HEPES-buffered medium supplemented with 10% FBS and the dish was transferred to a 37 °C incubator. The cells were washed once with 37 °C HEPES-buffered medium and then incubated with DMSO or inhibitors prepared in HEPES buffered medium for 1 h at 37 °C. The treatments were started with 20 min intervals for the four wells, and following the last addition of the inhibitor/DMSO solution, the dish was transferred to a sample chamber at the LSM 780 microscope. The temperature at the chamber was kept at 37 °C. To stain endosomes, the cells were washed once with Live Cell Imaging Solution (A14291DJ, ThermoFisher Scientific) and then 5 µg/ml transferrin labelled with Alexa647 (transferrin-A647; T-23366, Invitrogen) prepared in Live Cell Imaging Solution and containing inhibitor/DMSO was added to cells and incubated for 1 min. To stain cell membranes with the environment-sensitive membrane probe NR12S, NR12S was diluted in Live Cell Imaging Solution and immediately added to cell medium resulting in a final probe concentration of 20 nM. Imaging was started 10 min after the addition of NR12S and continued for up to 20 min resulting in 5-7 images per condition. The images were acquired using a Zeiss LSM 780 microscope with pixel size of 0.13 µm and a pinhole of 50 µm. The focus was set to the equatorial plane of the cells slightly above the cover glass surface. NR12S was excited using the 514 nm laser line and two images were acquired simultaneously at 520-580 nm and 584-644 nm. Then, the image for the transferrin staining was acquired using excitation with the 633 nm laser line and detection at 650-735 nm. This imaging order was used to minimize photo-bleaching of the NR12S probe.

To quantify the generalized polarization (GP) value, the two images acquired for NR12S staining were analyzed using an ImageJ macro adapted from [3], and the GP value was calculated:

$$GP = \frac{I_{520-580} - I_{584-644}}{I_{520-580} + I_{584-644}}$$

To quantify a GP value specifically at the plasma membrane, the plasma membrane area was manually marked in the cell. For the endosome selection, the cytoplasm area was manually marked in the cell and then automatic thresholding (“Otsu” function in Fiji) was used to select pixels positive for transferrin within the marked area. Finally, the created plasma membrane and endosome masks were overlaid with the GP image obtained from the ImageJ macro, and mean GP values within the plasma membrane and endosome masks were calculated. In the calculation, only the pixels with intensity values above threshold levels in both channels for NR12S were included.

**References**

1. Bumpus TW, Baskin JM (2017) Clickable Substrate Mimics Enable Imaging of Phospholipase D Activity. ACS Central Science 3 (10):1070-1077. doi:10.1021/acscentsci.7b00222

2. Kotecha N, Krutzik PO, Irish JM (2010) Web-based analysis and publication of flow cytometry experiments. Curr Protoc Cytom Chapter 10:Unit10 17. doi:10.1002/0471142956.cy1017s53

3. Owen DM, Rentero C, Magenau A, Abu-Siniyeh A, Gaus K (2011) Quantitative imaging of membrane lipid order in cells and organisms. Nat Protoc 7 (1):24-35. doi:10.1038/nprot.2011.419

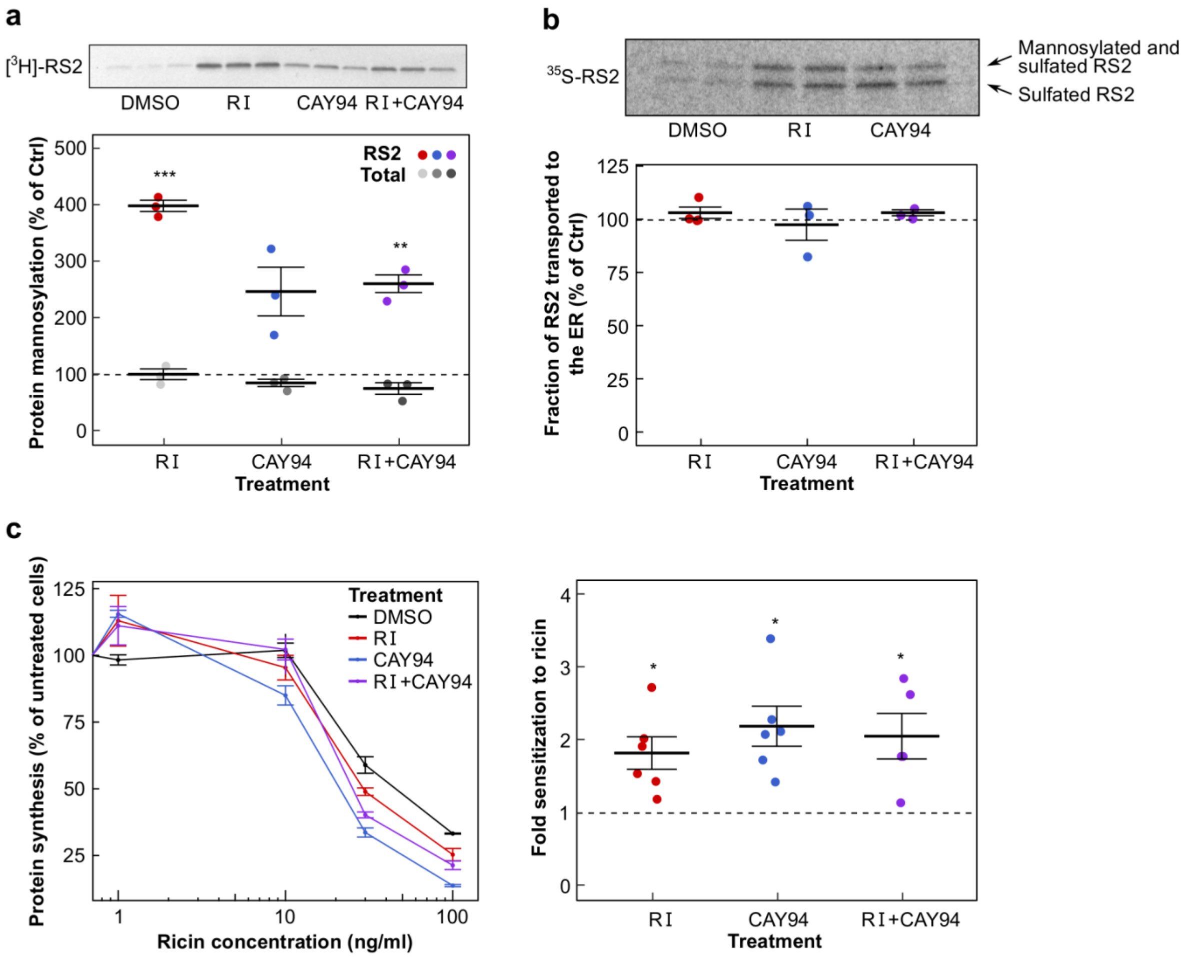


**Fig. S1 DGK and PLD inhibition increase ricin transport to the ER and cytosol**

HEp-2 cells were subjected to (a) mannosylation and (b) sulfation assays with ricin-sulf2 (RS2) after incubation with 10 µM RI and/or 10 µM CAY94 for 1 h. A representative autoradiograph is shown. For (a), quantification of both toxin mannosylation and total protein mannosylation is shown, *n*=3. For (b), the fraction of sulfated ricin reaching the ER was determined (upper band, size shift due to glycosylation) and results from 4 experiments are shown. (c,d) HEp-2 cells were treated with 10 µM of inhibitors for 1 h and subsequently incubated with increasing concentrations of ricin (1, 10, 30 and 100 ng/ml) for 3 h, before protein synthesis was measured. One representative experiment is shown in (c) and the sensitization to ricin from 6 experiments (d) is shown.

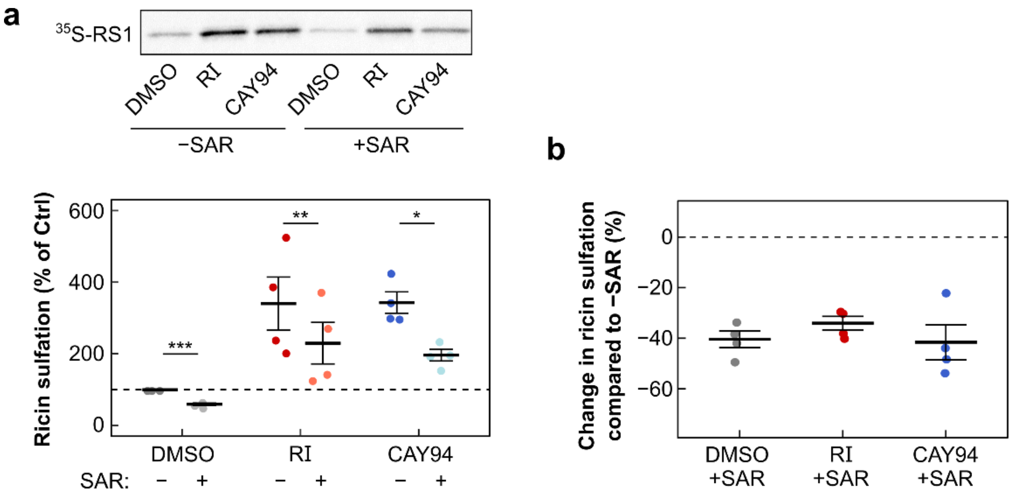


**Fig. S2 The Vps34 inhibitor SAR405 reduces ricin sulfation**

HEp-2 cells were subjected to sulfation assay with ricin-sulf1 (RS1) after treatment with 0.1% DMSO (control), 10 µM RI or 10 µM CAY94 in the presence or absence of the Vps34 inhibitor SAR405 (SAR; 10 µM) for 1 h. (a) Representative autoradiogram from one representative experiment and the quantifications of RS1 sulfation expressed as percent of control (*n*=4). (b) SAR-mediated reduction in ricin sulfation expressed as percent change compared to the sample without SAR.

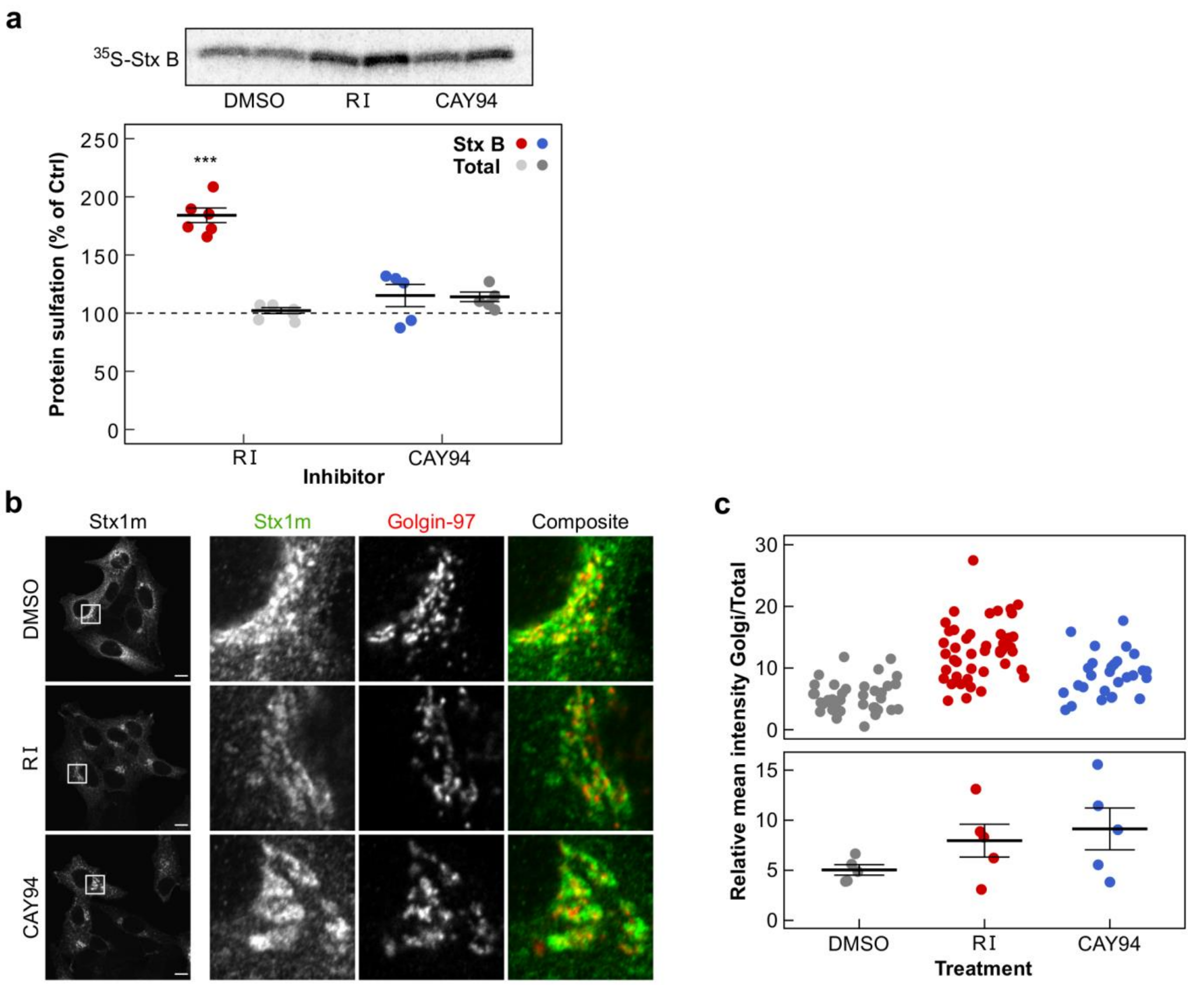


**Fig. S3 Shiga toxin transport to the Golgi is increased upon DGK and PLD inhibitor treatment**

(a) HEp-2 cells were subjected to sulfation assay with StxB-sulf2 after treatment with the 10 µM of the indicated inhibitors for 1 h. The upper figure shows an autoradiogram from a representative experiment and the lower figure shows the quantification of StxB-sulf2 sulfation expressed as percent of control ($n \geq 5$). (b, c) HEp-2 cells were treated with 0.1% DMSO, 10 µM RI or 10 µM CAY94 for 1 h prior to addition of Stx1m (final conc. 100 ng/ml). After 1 h, the cells were fixed, permeabilized, immunolabelled for Stx (green) and golgin-97 (red), and mounted with ProLong Gold. (b) Representative confocal images; scale bar, 10 µm. (c) To quantify Stx1m transport to the Golgi, at least 20 individual cells from 6 - 10 confocal images were manually marked and analyzed using Fiji. To adjust for variation in the levels of Stx1m associated to individual cells, mean Stx1m labeling in the Golgin-97 positive structures (Golgi) was divided by the mean intensity of the Stx1m outside the Golgi. The upper figure shows quantitative data from one representative experiment and the lower figure shows the summary of means from 5 independent experiments.

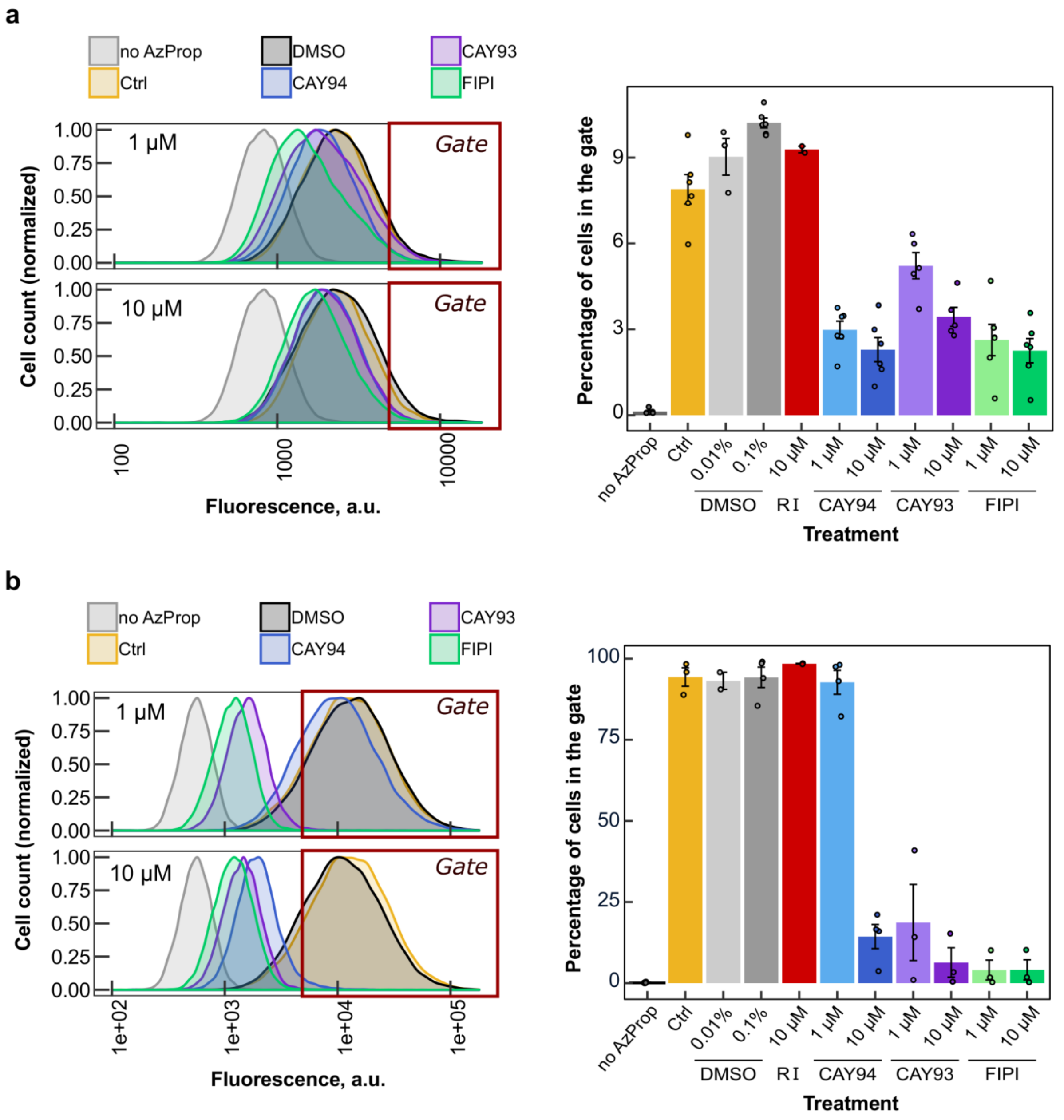


**Fig. S4 PLD activity in HEp-2 cells**

(a) Basal and (b) PMA-induced PLD activity in HEp-2 cells was measured using the IMPACT assay. The cells were treated with indicated inhibitors for 1 h before incubating with 0.5 µM AzProp for (a) 2 h or (b) 30 min which was followed by 30 min incubation with 0.1 µM PMA for stimulated PLD activity (only b). Then, the cells were incubated with 0.5 µM BDP FL DBCO for 10 min, detached by Accutase, fixed, washed in 0.05% saponin with 1 % BSA and analyzed by flow cytometry. Single cells were gated based on forward and side scattering. To quantify the percentage of the cells with high PLD activity, a manual gate was set to get 10% of the cells with highest BDP FL DBCO signal in the sample treated with 0.1% DMSO. The percentage of the cells in this gate was then quantified for all other conditions. *Left:* representative data from a single experiment. *Right:* Mean percentage of the cells in the gate ± SEM ($n$=3-5; except for RI: $n$=2).

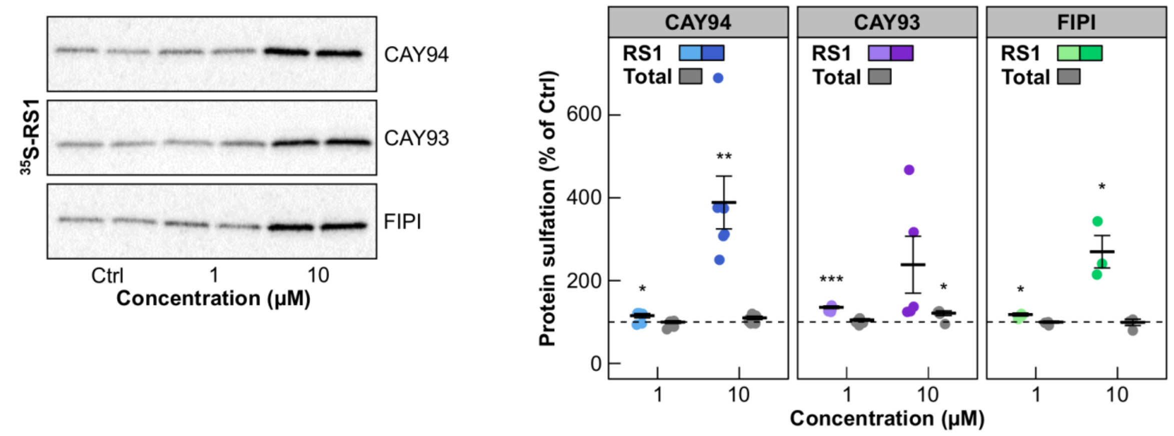


**Fig. S5 Ricin sulfation after treatment with different concentrations of PLD inhibitors**

HEp-2 cells were subjected to sulfation assay with RS1 after treatment with the 1 or 10 µM of the indicated inhibitors for 1 h. *Left*: Autoradiograms from representative experiments. *Right:* Quantification of RS1sulfation and total protein sulfation expressed as percent of control (*n*≥3).

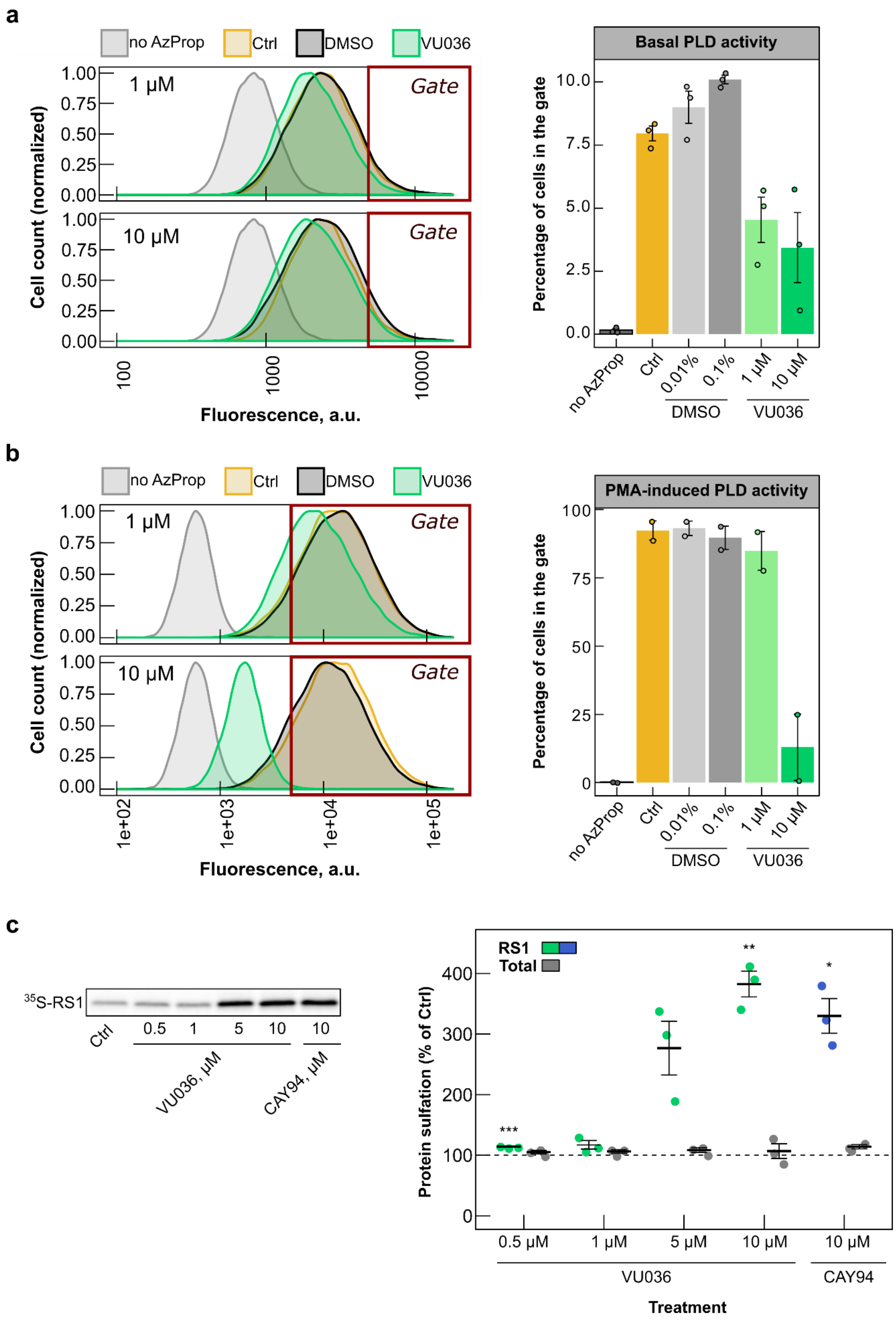


**Fig. S6 PLD activity and ricin sulfation after treatment with the PLD inhibitor VU036**

*(continued on the next page)*

(Fig. S6) (a) Basal and (b) PMA-induced PLD activity in HEp-2 cells was measured using the IMPACT assay. The cells were treated with DMSO or VU036 for 1 h before incubating with 0.5 µM AzProp for (a) 2 h or (b) 30 min which was followed by 30 min incubation with 0.1 µM PMA for stimulated PLD activity (only b). Then, the cells were incubated with 0.5 µM BDP FL DBCO for 10 min, detached by Accutase, fixed, washed in 0.05% saponin with 1 % BSA and analyzed by flow cytometry. Single cells were gated based on forward and side scattering. To quantify the percentage of the cells with high PLD activity, a manual gate was set to get 10% of the cells with highest BDP FL DBCO signal in the sample treated with 0.1% DMSO. The percentage of the cells in this gate was then quantified for all other conditions. *Left:* representative data from a single experiment. *Right:* Mean percentage of the cells in the gate ± SEM, (a) *n*=3 and (b) *n*=2. (c) HEp-2 cells were subjected to sulfation assay with RS1 after treatment with 0.5 µM, 1 µM, 5 µM or 10 µM of VU036, or with 10 µM of CAY94 for 1 h. *Left*: Autoradiogram from a representative experiment. *Right:* Quantification of RS1sulfation and total protein sulfation expressed as percent of control, *n*=3.

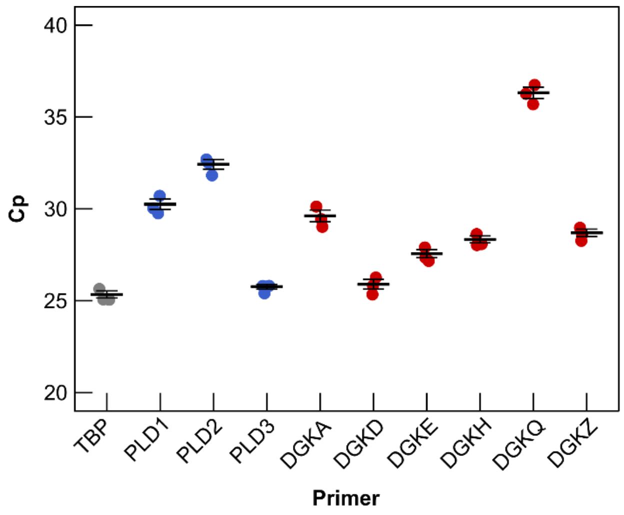


| Primer | Efficiency | Std. error |
|---|---|---|
| TBP | 1.980 | 0.011 |
| PLD1 | 1.882 | 0.063 |
| PLD2 | 1.639 | 0.063 |
| PLD3 | 1.974 | 0.020 |
| DGKA | 1.951 | 0.027 |
| DGKD | 2.020 | 0.056 |
| DGKE | 2.076 | 0.024 |
| DGKH | 1.948 | 0.008 |
| DGKQ | 1.971 | 0.303 |
| DGKZ | 1.940 | 0.013 |

**Fig. S7 PLD and DGK isoforms in HEp-2 cells.**

Total RNA was isolated from HEp-2 cells, cDNA was synthesized and analyzed by qPCR using primers for different PLD and DGK isoforms: PLD1, PLD2, PLD3, DGKα (DGKA), DGKδ (DGKD), DGKε (DGKE), DGKη (DGKH), DGKθ (DGKQ), DGKζ (DGKZ); TBP was used as a reference gene. The figure shows Cp values for different primers (the dots represent 3 independent RNA isolations that were analyzed together by qPCR). Primer efficiencies were obtained by running a standard curve for each primer.

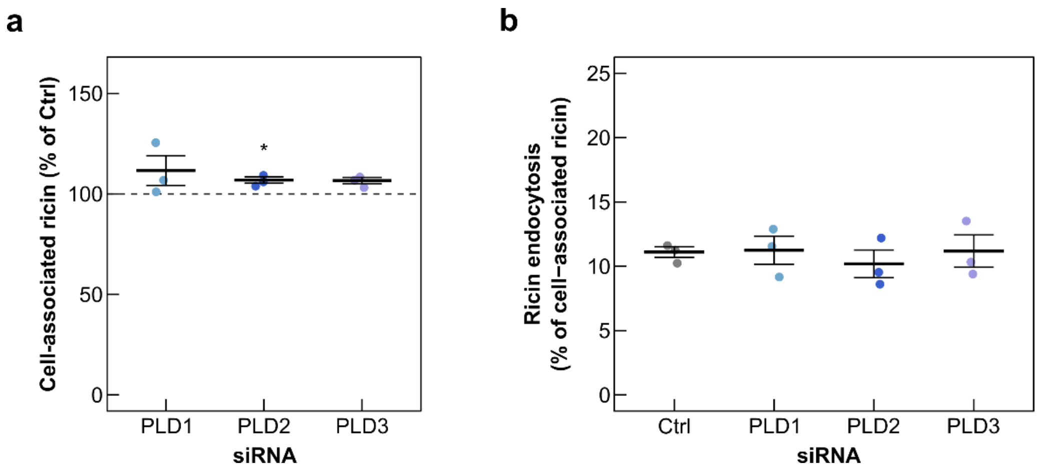


**Fig. S8 PLD knockdown does not inhibit the binding or endocytosis of ricin.**

HEp-2 were transfected with 10 nM siRNA against PLD1, PLD2 o PLD3, or with 10 nM control siRNA for 48 h, followed by incubation with $^{125}$I-ricin for 20 min to measure ricin endocytosis. (a) Total cell-associated ricin expressed as percent of control; (b) Endocytosed ricin expressed as percent of total cell-associated ricin in each condition; *n*=3.

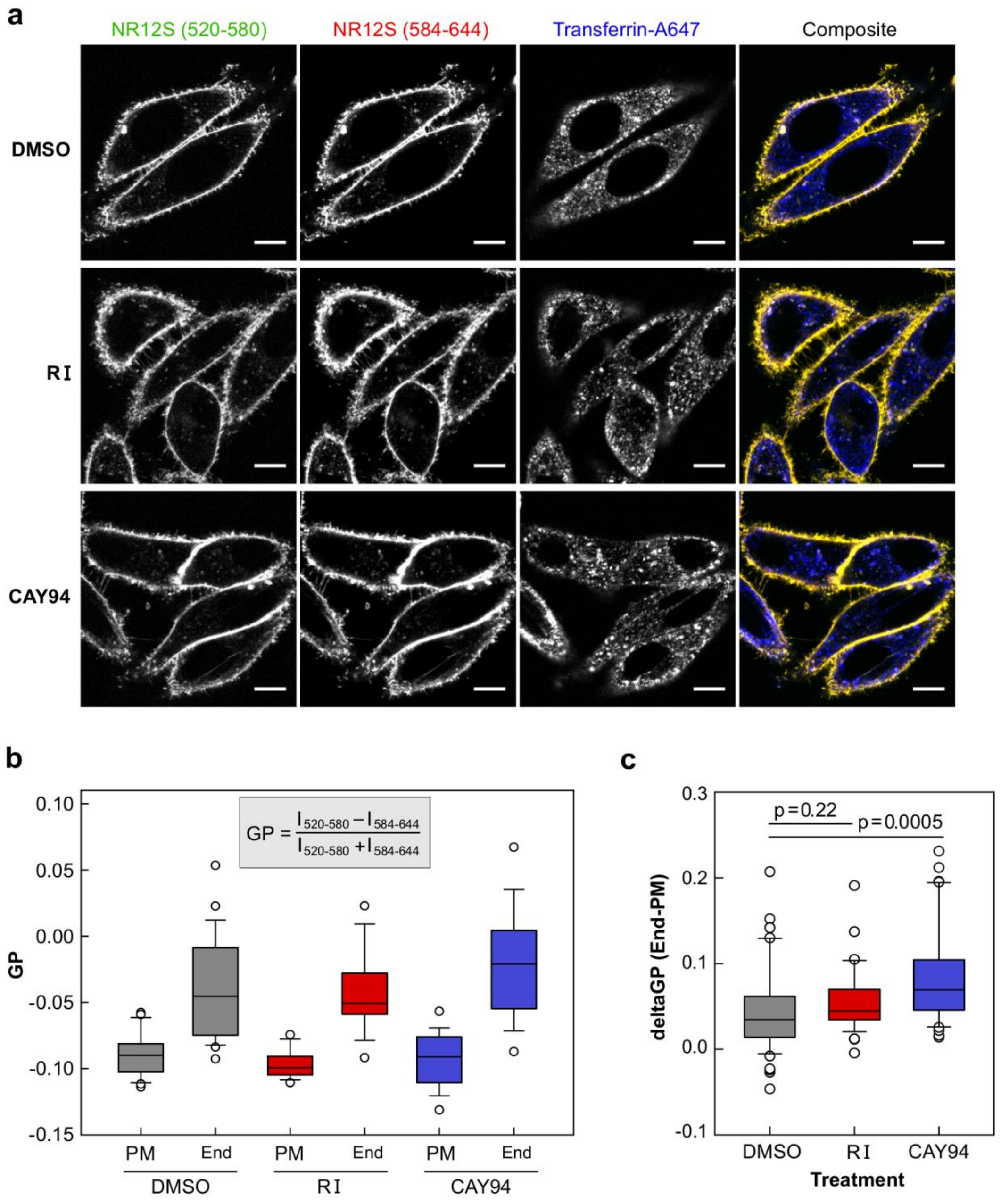


**Fig. S9 Membrane packing in endosomes and the plasma membrane after inhibitor treatment**

HEp-2 cells were treated with 10 µM RI, 10 µM CAY94 or 0.1% DMSO for 1h, prior to addition of transferrin-A647 and staining with 20 nM NR12S. Two images (520-580 nm and 584-644 nm) were acquired simultaneously for the NR12S dye and were used to quantify a GP value for each pixel above the threshold. Sample images are shown in (a); scale bar, 10 µm. To quantify the mean GP value in the endosomes, a manual selection of the cell cytoplasm was combined with a thresholded image from the transferrin-A647 channel to create an endosome mask (pixels positive for transferrin signal within the cytoplasm). To quantify the mean GP value in the plasma membrane, a plasma membrane mask was created by manual selection. (b) Mean GP values for transferrin-positive endosomes (End) and the plasma membrane (PM). The figure shows data from one of two independent experiments (19-22 cells/condition). (c) The difference between GP values in the transferrin-positive endosomes and the plasma membrane for each cell (deltaGP). The figure shows pulled data from two independent experiments (36-54 cells/condition).

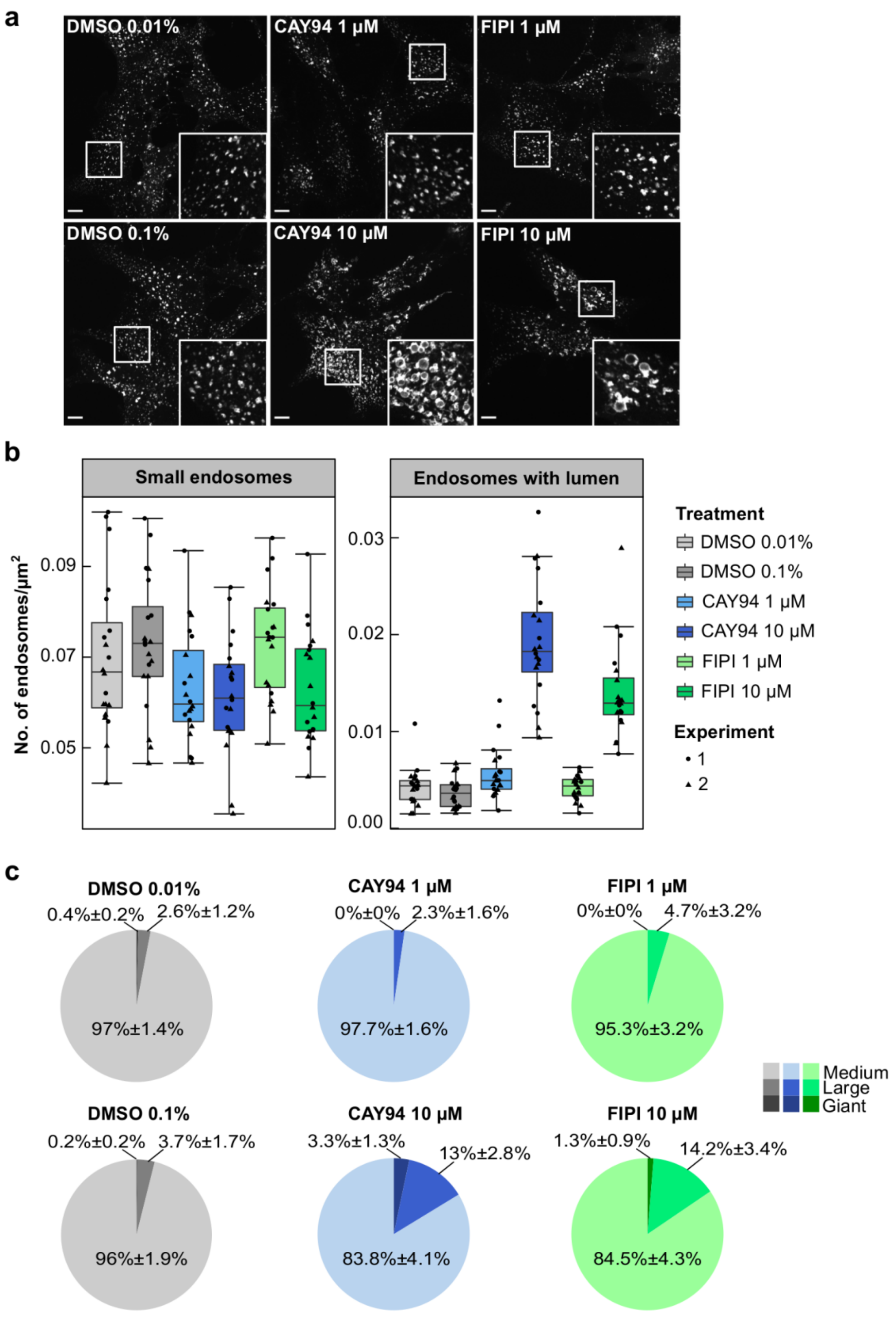


**Fig. S10 Endosome size after treatment with different concentrations of CAY94 and FIPI.**

RPE GFP-WDFY2 cells were treated with the inhibitors for 1 h, fixed, mounted and imaged by a confocal microscope. (a) Representative images of GFP-WDFY2 positive endosomes; scale bar, 10 µm. Ten images per condition from two independent experiments were analyzed as described for Figure 5. (b) The number of small endosomes and the number of endosomes with lumen detected per cell area. (c) Distribution of medium, large and giant endosomes within the subpopulation of endosomes with detected lumen. Mean values ± difference between the two experiments.
